# Deep CNN ResNet-18 based model with attention and transfer learning for Alzheimer's disease detection

**DOI:** 10.3389/fninf.2024.1507217

**Published:** 2025-01-07

**Authors:** Sofia Biju Francis, Jai Prakash Verma

**Affiliations:** ^1^Department of Computer Science and Engineering, Institute of Technology, Nirma University, Gujarat, India; ^2^Department of Computer Engineering, NMIMS, MPSTME, Mumbai, India

**Keywords:** Alzheimer's disease, cognitive deterioration, ResNet-18, depth convolution, squeeze and excitation, transfer learning

## Abstract

**Introduction:**

The prevalence of age-related brain issues has risen in developed countries because of changes in lifestyle. Alzheimer's disease leads to a rapid and irreversible decline in cognitive abilities by damaging memory cells.

**Methods:**

A ResNet-18-based system is proposed, integrating Depth Convolution with a Squeeze and Excitation (SE) block to minimize tuning parameters. This design is based on analyses of existing deep learning architectures and feature extraction techniques. Additionally, pre-trained ResNet-18 models were created with and without the SE block to compare ROC and accuracy values across different hyperparameters.

**Results:**

The proposed model achieved ROC values of 95% for Alzheimer's Disease (AD), 95% for Cognitively Normal (CN), and 93% for Mild Cognitive Impairment (MCI), with a maximum test accuracy of 88.51%. However, the pre-trained model with SE had 93.26% accuracy and ROC values of 98%, 99%, and 98%, while the model without SE had 94%, 97%, and 94% ROC values and 92.41% accuracy.

**Discussion:**

Collecting medical data can be expensive and raises ethical concerns. Small data sets are also prone to local minima issues in the cost function. A scratch model that experiences extensive hyperparameter tuning may end up being either overfitted or underfitted. Class imbalance also reduces performance. Transfer learning is most effective with small, imbalanced datasets, and pre-trained models with SE blocks perform better than others. The proposed model introduced a method to reduce training parameters and prevent overfitting from imbalanced medical data. Overall performance findings show that the suggested approach performs better than the state-of-the-art techniques.

## 1 Introduction

**Figure 1:**
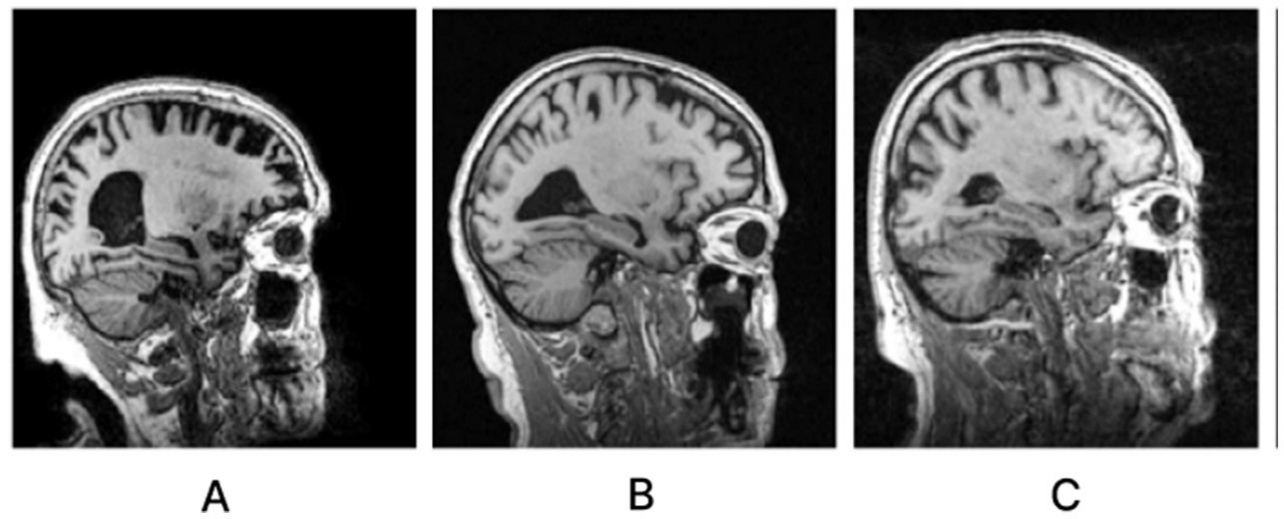
Sagittal middle slice of **(A)** Alzheimer's disease (AD), **(B)** Mild cognitive impairment (MCI), **(C)** Cognitively normal (CN).

Alzheimer's disease (AD) is a severe neurological condition. A person with AD is unable to converse, retain details, make decisions, pick up new skills, and so on (Korolev, 2014; Hazarika et al., 2023). The majority of people with Alzheimer's disease are elderly or in their early 60s. The most catastrophic of all the physical alterations is damage to brain cells. The brain areas that sustain the most significant damage are the amygdala, hippocampus, and a few additional areas that control most AD symptoms. The patient is unable to perform even the most basic tasks because learning cells are first impacted, then additional gray matter cells are destroyed. Consequently, those suffering from Alzheimer's disease experience extreme behavioral and cognitive challenges in addition to memory loss. The majority of individuals with AD have advanced from Mild Cognitive Impairment (MCI), an early stage of dementia. The symptoms of MCI are nearly the same as those of AD, albeit less severe. MCI is sometimes called AD in its early stages. Research indicates that eight out of ten individuals with MCI acquire AD within 7 years (Hazarika et al., 2023). Typically, neuro-specialists work alongside psychologists to administer various mental and physical examinations, including a Mini-Mental State Examination (MMSE), a neurophysiological assessment, a physical assessment and screening tests, a depression analysis, and more. Various instruments are needed to complete these tasks, making the procedure inefficient and time-consuming. To provide tissue-by-tissue information on the neurological system, Magnetic Resonance Imaging (MRI) is a widely used method. Compared to the conventional diagnosis method, brain imaging may require less time and equipment for AD classification. Furthermore, proper brain imaging processing may identify significant biomarkers long before the beginning of Alzheimer's disease (Hazarika et al., 2023). Conversely, intricate pixel formations make diagnosing AD difficult by looking at tissue changes for traditional image processing methods (Fung et al., 2019). Sample brain MRI scans for patients with AD, MCI, and Cognitively Normal (CN) are shown in Figure 1.

The intricacy of Alzheimer's disease and the necessity of timely identification make it a formidable medical issue. Conventional techniques might not always be enough to predict the course of the illness with enough accuracy. Alzheimer's is a complicated medical condition that requires prompt diagnosis due to its complexity. Deep Learning (DL) techniques are increasingly being used by medical experts to visualize and predict diseases and create individualized, preemptive treatment regimens (Saleh et al., 2023). Traditional Machine Learning (ML) and DL algorithms aimed to solve discrete, highly data-intensive jobs. Transfer learning has emerged as a solution to the isolated learning paradigm to improve classification performance using MRI scans. Its goal is to use the characteristics learned from pre-trained models. Creating effective models to help radiologists and medical professionals recognize the various phases of Alzheimer's disease, such as AD, MCI, and CN.

Francis et al. (2024) employed a deep learning model to evaluate the ADNI dataset and identify the stages of Alzheimer's disease using Squeeze and Excitation Networks (SENet) and local binary patterns. The accuracy of classifying MCIc (MCI converter) against MCInc (MCI non-converter) was 86% with SE networks and 82% without SE networks. Lu et al. (2022) developed a two-stage model that used contrastive learning and transfer learning (ResNet) to predict the progression of MCI into AD, with an accuracy of 82% and an AUC of 84%. The improvised model ADNet was created by integrating SENet to VGG-16 and improving feature extraction after evaluating the ADNI 2D MRI images. Without SENet, the accuracy in AD vs. CN classification was 82.94%, but with SENet, it was 84.08%. By developing an optimal weighted ensemble model consisting of five pre-trained 3D CNN ResNet-50 variants (ResNet, ResNeXt, SEResNet, SEResNeXt, and SE-Net), Dharwada et al. (2024) concentrated on early AD diagnosis using sMRI images from the ADNI dataset and achieved an accuracy of 97.27%. Illakiya et al. (2023) developed a hybrid attention model to extract both global and local features by utilizing 3D MRI from ADNI. It achieved an accuracy of 89.17% with DenseNet-169 along with SENet. With DenseNet-169 alone, able to reach 77% accuracy. Khan et al. (2022) suggested PMCAD-Net, an applicable multiclass classification network for the early detection of Alzheimer's disease. Using the ADNI dataset, it distinguishes between EMCI, LMCI, CN, and AD, achieving a 98.9% accuracy rate and a 96.3% F1 score. Khan et al. (2023) proposed a transfer learning-based method (VGG-16 and VGG-19) that differentiated between CN, EMCI, LMCI, and AD by using tissue segmentation to extract gray matter from the ADNI dataset's MRI images and achieved an accuracy of 97.89%.

Odusami et al. (2021) was able to analyze fMRI images from ADNI and predict the early stages of AD with an accuracy of 80.80% for AD vs. CN categorization by unfreezing all layers of ResNet-18 and modifying all parameters to fit the new dataset. The accuracy of the ensemble model developed from the analysis of fMRI images from ADNI was 97.9%, 87.5% with ResNet-18, and 96.2% with VGG-16 in Tajammal et al. (2023). Utilizing an attention mechanism, ResNet-18, Zhou et al. (2023) developed a 2D parameterized model. While average pooling produces an accuracy of 88.12%, ResNet-18 alone produces an accuracy of 82.45%. A ROC of 98.49% is provided by the complete model. Suja and Raajan (2024) evaluated a number of deep learning models for the timely diagnosis of AD using the ADNI dataset. The accuracy of the ResNet-18 model with RMSprop and ADAM was 83.5%. Topsakal and Lenkala (2024) proposed an ensemble model that used a pre-trained, improved model with gradient boosting to attain a 95% accuracy rate. However, ResNet-50 alone had an accuracy of 88%.

However, graph convolutional networks can analyze the pathological brain regions associated with cognitive disorders and achieve good classification performance. They cannot uncover the fundamental relationships between multiple brain ROIs associated with illness. These networks first retrieve characteristics for each ROI or subject before building a classifier for AD diagnosis using either unimodal or bimodal imaging data. Utilizing hypergraph-based techniques, which consider high-order relations between numerous ROIs from unimodal or multimodal imaging, improved classification performance and created discriminative connections.

Zuo et al. (2024) designed a Graph-based model and forecast aberrant brain connections at various AD stages. The prior distribution from graph data is estimated using a Kernel Density Estimation (KDE) technique, and an adversarial learning network is integrated for multimodal representation learning. The Pairwise Collaborative Discriminator (PCD) preserves sample and distribution consistency, and a hypergraph-based network is developed to fuse the learned representations to produce united connectivity-based features. For the binary classification (AD vs. CN, LMCI vs. CN, and EMCI vs. CN), 300 individuals were gathered from the ADNI-3 database for the investigation. The accuracy rates were 96.47%, 92.20%, and 87.50%, respectively. Pan et al. (2024) developed a comprehensive system integrating fMRI and DTI to identify aberrant brain networks for AD research. This study used 236 ADNI subjects and obtained an accuracy of 80.72% for CN, 63.8% for EMCI, 86.91% for LMCI, and 82.71% for AD. Zong et al. (2024) constructed brain networks using Diffusion-based Graph Contrastive Learning (DGCL) to identify the geographical positions of brain regions precisely. Contrastive learning is adopted to optimize brain connections by removing individual differences in redundant connections unrelated to disorders. Data (DTI format) from 349 subjects-including those in the Control Normal group (CN), Early Mild Cognitive Impairment (EMCI), Late Mild Cognitive Impairment (LMCI), and AD were gathered from the ADNI, and an accuracy of 86.61% was produced. Zuo et al. (2023) presented a brain structure-function fusing learning model by fusing representation from rs-fMRI and DTI to investigate MCI. The knowledge-aware transformer module automatically captures features of local and global connections across the brain. Achieved an accuracy of 87.80% for EMCI vs. Subjective Memory Complaints (SMC), 95.57% for LMCI vs. SMC, and 91.14% for LMCI vs. EMCI.

Every deep learning network that is constructed from the ground up will run into a lot of issues. A Deep CNN network requires a large number of parameters to be trained during the training phase. By lowering this parameters, the network's computational complexity and time and space constraints would decrease. For the same reason, we can use Squeeze and Excitation blocks to minimize the total training parameters, identify the channel interdependencies, and recalibrate the output feature with input to exploit the local and global spatial features to improve the performance. We can also use Depth Convolution instead of standard Convolution. The fully connected layer, including the dropout layer, can be replaced with Global Average Pooling to lower the overall training parameters and accelerate convergence. This regularization technique significantly reduces overfitting and generalization errors in the model. However, an extensive dataset for model training and meticulous hyperparameter optimization for increased efficiency are no longer necessary when employing the Transfer Learning technique. The main contributions of our work are as follows.

- Squeeze and Excitation (SE) blocks were added to the ResNet-18 residual block in order to build a Deep CNN ResNet-18-based model from the ground up using the attention mechanism. Additionally, to lower training parameters and lighten the model overall, we employed Depth-wise Convolution and Global Average pooling. ROC and accuracy were compared to various combinations of optimizers, dropout rates, sample sizes, and epochs using the ADNI dataset, which was utilized to train the model. Cross-validation is used to validate the model and lower the regularization errors.
- The accuracy and ROC were assessed through training and testing using the pre-training model ResNet-18 with SE block and ADNI dataset.
- Through training and testing with the pre-training model ResNet-18 without SE block, the accuracy and ROC were evaluated using the ADNI dataset.
- ROC values, testing and training accuracy, confusion matrix, and other evaluation parameters were used to compare the performance of the three models mentioned above.
- The models were assessed using real-time patient MRI scans along with their demographic data as well as with the OASIS-1 dataset.

Medical professionals are using deep learning for disease prediction and visualization to create personalized treatment plans (Saleh et al., 2023). In comparison to the three deployed models and the state-of-the-art techniques outlined, ResNet-18, a pre-trained model with SE block, demonstrated the most convincing performance.

Five distinct sections that make up the overall paper. An outline of Alzheimer's disease, the motivation behind this effort, and a survey summary of associated works are covered in Section 1. Section 2 describes the dataset, data pre-processing, and proposed model's architectures in detail. The results are demonstrated and compared using several criteria in Section 3. Lastly, the conclusion, limitations, and future work are discussed in Sections 4 and 5.

## 2 Materials and methods

**Figure 2:**
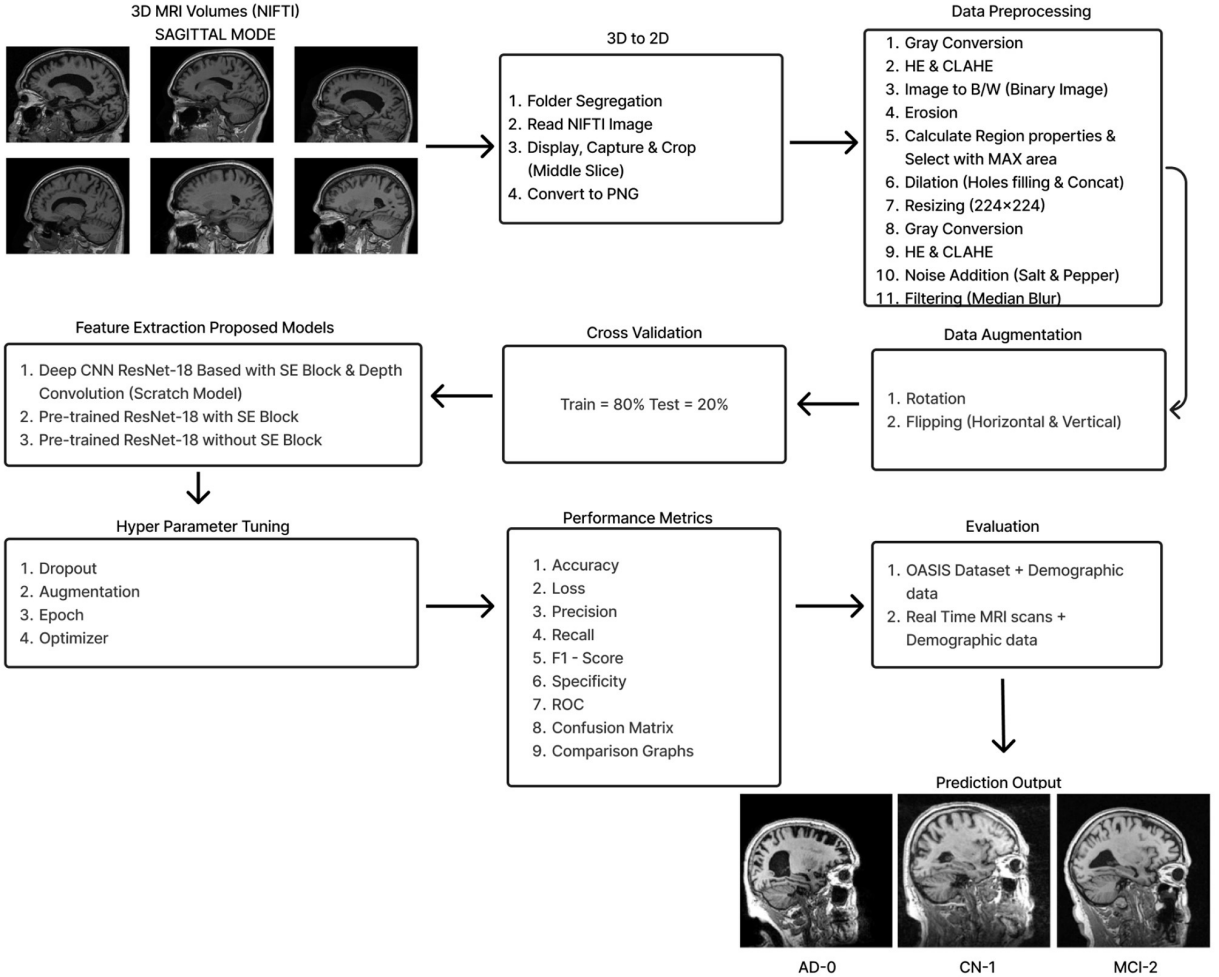
Overall procedure.

The initial section outlines the datasets utilized for training and evaluating all the models, along with a detailed description of the preprocessing steps that make these datasets suitable for both training and testing. The following section explains the functionality of the Deep CNN architecture based on ResNet-18, which was developed from scratch. This architecture incorporates depthwise convolution and the Squeeze and Excitation algorithms. Additionally, it reviews the framework of two other models that employ a transfer learning approach. Figure 2 shows the complete procedure of our suggested methodology.

### 2.1 Dataset

This section covers the datasets used for training and testing, along with details about the preprocessing methods applied to 8-bit images available in each dataset.

#### 2.1.1 ADNI

The ADNI (Alzheimer's Disease Neuroimaging Initiative) database (http://adni.loni.usc.edu/ accessed on August 2022) provided the study's data. These comprised the core of our analysis: volumetric T1-weighted, B1 corrected, N3 scaled, Magnetization-Prepared Rapid Gradient Echo (MP-RAGE) MRIs with Gradwarp. Alzheimer's Dementia (AD), Cognitively Normal (CN), and Mild Cognitive Impairment (MCI) were the three categories of the dataset that comprised the 2291 (476-AD, 703-CN, 1112-MCI) patients in this study. It is 46.83 GB compressed and 70.8 GB uncompressed and contains MRI images in NIFTI format. A single NIFTI image contains a packed volume of ~184 slices.

#### 2.1.2 Real-time hospital patient data

MRI imaging data from eight patients at the Government Medical College in Kottayam, Kerala, were collected for model evaluation. Each patient had around 250–400 DICOM images converted to JPG format. From these, six to nine sagittal-mode JPG images were randomly selected for pre-processing. They also provided the manually conducted tests for the MMSE and the CDR, along with their corresponding scores.

#### 2.1.3 OASIS-1

OASIS-1 (Open Access Series of Imaging Studies) is a publicly accessible dataset used to test our approach. It is 15.8 GB compressed and 50 GB uncompressed and available at http://www.oasis-brains.org. Twelve archive files are available for download, organized by imaging session. Each session directory contains an XML file, a TXT file, and three subdirectories: RAW, PROCESSED, and FSL_SEG. The XML and TXT files provide acquisition and anatomical measurements, while the RAW directory holds the individual scan images. The PROCESSED directory includes two subdirectories: SUBJ_111 and T88_111. The SUBJ_111 folder contains averaged and co-registered sagittal images in GIF format. The T88_111 folder has the atlas-registered, gain field-corrected image and its brain-masked version for all three projections, also in GIF format. The FSL_SEG directory contains the segmentation image for gray matter, white matter, and Cerebro Spinal Fluid (CSF) generated from the masked atlas image. The “oasis_cross-sectional.csv” spreadsheet contains labels for demographics, clinical information, and derived anatomic volumes. Ten randomly selected sagittal GIF images were converted to JPG format for applying our various pre-processing algorithms, enabling model testing.

#### 2.1.4 Data preprocessing

All three datasets were mainly preprocessed using MATLAB. For the ADNI dataset, the middle slice was carefully selected to observe the hippocampus region, which is the area most significantly affected by Alzheimer's disease (AD). Initially, the MRI images in NIFTI format were displayed and captured as a 2D image focusing on the middle slice using the screen capture function. The captured image was then cropped and saved in PNG format. Subsequently, the 2D images were organized into three distinct folders labeled AD, CN, and MCI, with assistance from the provided CSV file.

Preprocessing starts by converting the RGB image to grayscale to simplify the data and focus on intensity variations. Histogram Equalization is then applied to enhance contrast by stretching the pixel intensity histogram, improving detail visibility in different lighting conditions. Contrast_Limited_Adaptive Histogram Equalization (CLAHE) is used to boost contrast in low-contrast areas (Khalid et al., 2023). A binary image is created with a threshold of 0.3 to distinguish regions of interest from the background, followed by a few morphological operations to analyze the shapes and structures in the image. Erosion is an operation that reduces the size of an object by removing pixels from its boundaries. It utilizes a flat diamond-shaped structuring element with a distance of 3 units from the origin to the points of the diamond. After performing erosion, region properties are calculated, and the region with the maximum area is selected. Following this, morphological dilation is applied to restore the eroded regions and enhance the prominent features. The dilation uses a flat diamond-shaped structuring element with a distance of 8 units, as well as a disk-shaped element with a radius of 5 units. After dilation, morphological hole filling is used to address any gaps or holes within the segmented region. This process ensures that the area is complete and contiguous. As a result, the techniques applied yield a segmented image that clearly highlights the specific location of the disease within the Alzheimer's images, providing a precise visualization for further analysis.

The segmented image is resized to 224 × 224 pixels to ensure uniformity across the datasets, which facilitates efficient training. Subsequently, gray conversion, histogram equalization, and CLAHE are applied to simplify the data and further enhance image contrast. An impulsive noise, known as Salt-and-Pepper, is then introduced to the image to evaluate the robustness and effectiveness of the filtering technique. Finally, a compatible non-linear Median Blur filter technique is employed to remove the impulsive noise while preserving essential image details effectively.

During the preprocessing of the ADNI dataset, the following times were recorded for a single image:

- AD image: 0.9456 s for 2D conversion and 4.0455 s for preprocessing, totaling 4.9911 s.
- CN image: 0.9901 s for 2D conversion and 3.3949 s for preprocessing, totaling 4.385 s.
- MCI image: 1.2496 s for 2D conversion and 3.2866 s for preprocessing, totaling 4.5362 s.

**Figure 3:**
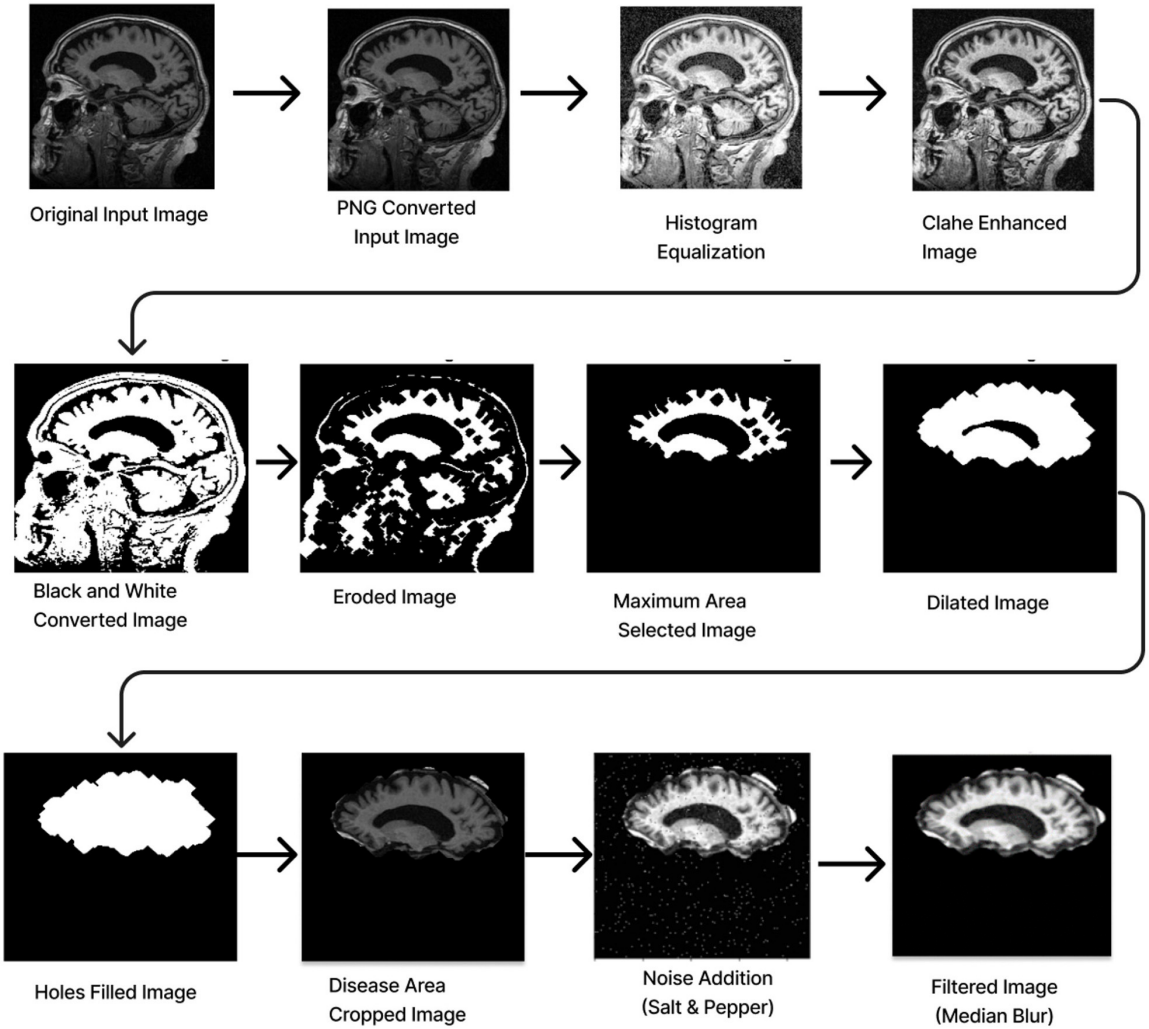
Image pre-processing.

The total preprocessing time for a single real-time image was 14.1287 s, while the OASIS-1 image took 12.6313 s. The complete path flow of image preprocessing is shown in Figure 3.

#### 2.1.5 Data augmentation

Although convolutional neural networks offer a great deal of generalization potential, this ability is severely constrained when the data set is small. This is due to the overfitting of the model. The problem of insufficient data could be efficiently addressed by utilizing data augmentation technology, which has been used to enhance the ADNI dataset using digital image processing technology (Gao et al., 2021). Owing to the restricted amount of data, the original dataset is supplemented by flipping horizontally, flipping vertically, and rotating. When an image is rotated, it is oriented around its center at a particular angle. A rotation of –15 to +15 is used in this investigation. The goal is to generate perspective adjustments from the same image so that models trained on more data can adapt better to changes in the orientation of the image's objects (Hasanah et al., 2023).

Data augmentation resulted in an expansion of the dataset from 2,291 images (476-AD, 703-CN, and 1,112-MCI) to 14,760 images (3,067:AD, 4,528:CN, 7,165:MCI) in the first stage and 25,357 images (5,267:AD, 7,779:CN, 12,311:MCI) in the second stage. This could perhaps solve the overfitting problem to some extent. In order to regularize the model and reduce the generalization error, the cross-validation techniques are then applied in an 80:20 ratio.

### 2.2 Deep CNN ReNet-18 based scratch model

This part will cover the Deep CNN ResNet-18-based model with the SE block and Depth Convolution in detail and explain each embedded block in the architecture.

#### 2.2.1 ResNet-18 pre-training model

**Figure 4:**
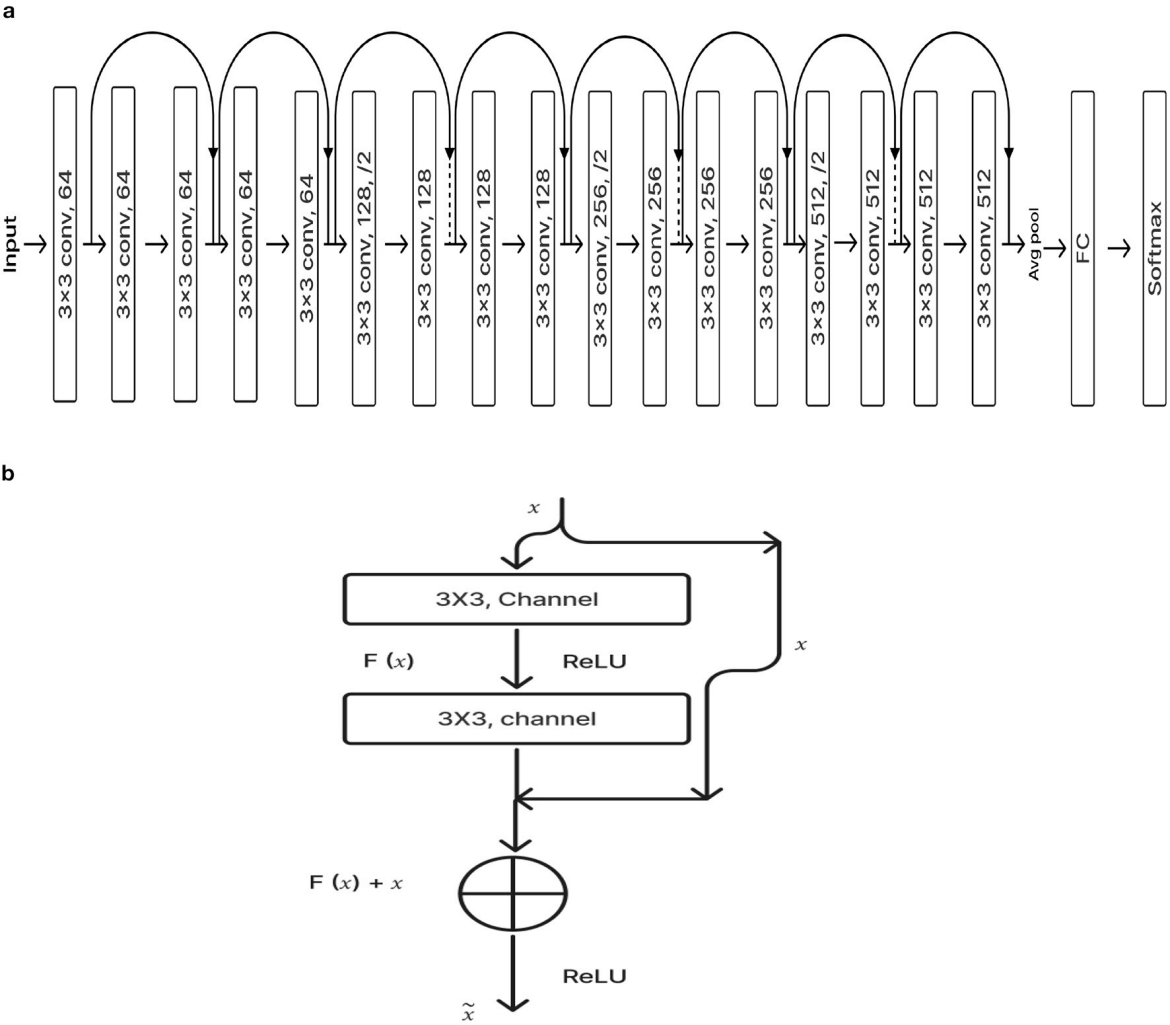
**(A)** ResNet-18 pre-training model. **(B)** Residual network.

ResNet-18 is composed of three layers: 17 convolution layers, a max pooling layer with a 3 × 3 filter size, and a fully connected layer. The block diagram for the pre-training ResNet-18 model is shown in Figure 4A. The ResNet-18 network is based on a residual building component (Gao et al., 2021). Figure 4B shows the structure of the residual building component.

After adding the input and output vectors directly through the convolutional layer, the result can be produced using the rectified linear unit (ReLU) activation function. This method can effectively address the issue of a deeper neural network's vanishing and exploding gradient. *F* stands for the residual function, while *x* and *y*, respectively, denote the input and output, which are referred to by Equation 1 (Odusami et al., 2021; Gao et al., 2021; Hasanah et al., 2023).


(1) $$\begin{array}{l} y=F\left(x\right)+x \end{array}$$

#### 2.2.2 Transfer learning

**Figure 5:**
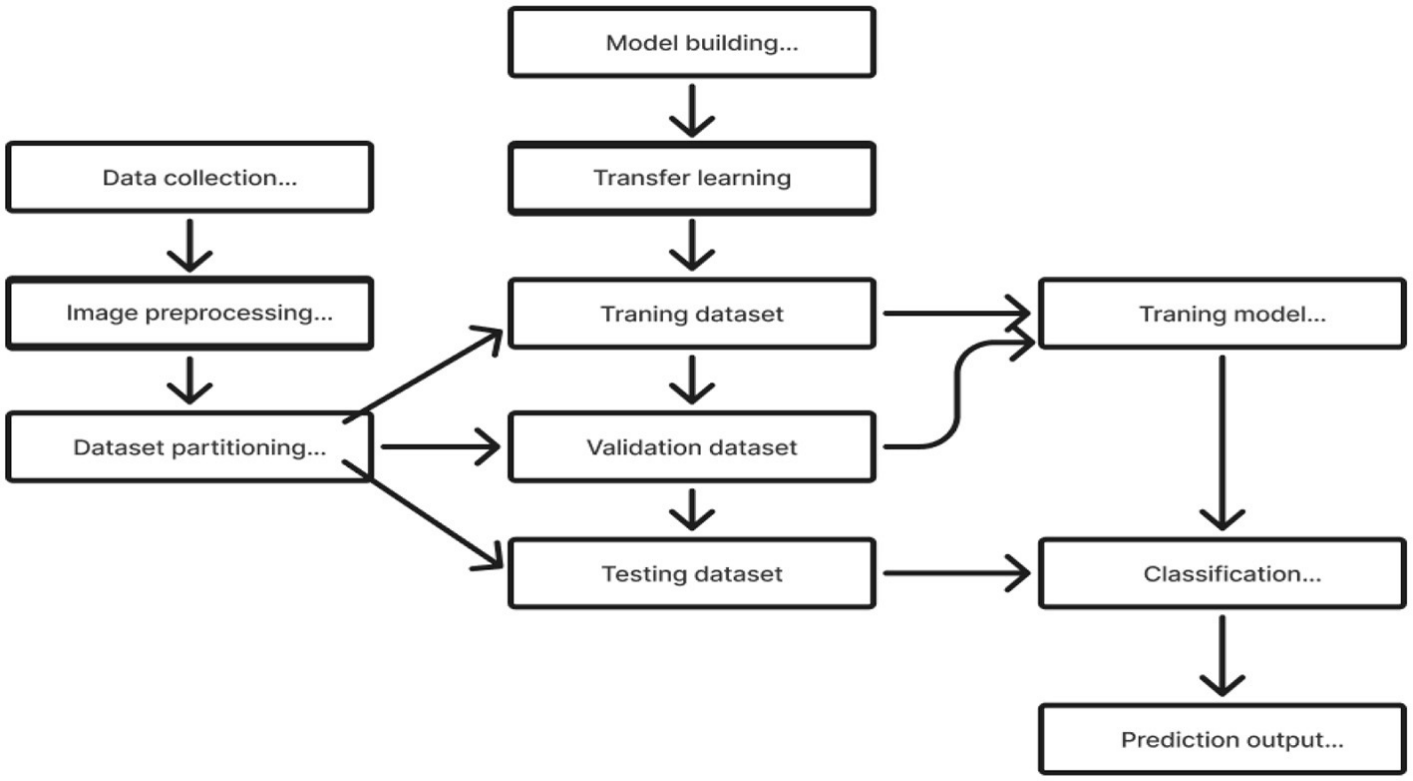
Transfer learning application.

Transfer learning is a useful technique for training deep learning (DL) models that leverage information from one domain to enhance performance on a different but related task or domain (Saleh et al., 2023). Pre-trained models are trained on large datasets, such as ImageNet, which comprises millions of images with over a thousand labels. As a feature extractor of general features, it is sensible and useful to apply the domain knowledge from these models to other domains, including medical classification tasks (Saleh et al., 2023). Due to the medical nature of the target data, we are unable to utilize the pre-trained model fully. We must address domain-specific difficulties in order to adjust the output layer. Reducing the number of training parameters needed to accomplish a task, increasing accuracy, and saving time are all achieved by using knowledge transfer models rather than randomly started models. This enables effective discrimination between the different classes in the dataset. In order to assist doctors in making precise and fast diagnoses, it first reduces the need for vast volumes of labeled data and offers a comprehensive and precise classification framework. This method solely trains the weights of the recently added layers for the particular task, freezing the weights of the previously trained model before training. This tactic lessens the problems brought on by a small and uneven dataset. The flow chart of our proposed model, which makes use of the Transfer Learning concept (Saleh et al., 2023; Gao et al., 2021), is shown in Figure 5.

#### 2.2.3 Global average pooling

The fully connected layer is a common classifier used by CNN. Training will be hindered and over-fitting is more likely due to the fully connected layer's excessive amount of parameters. Global average pooling, or GAP, is the procedure that generates the output by taking the global average of all the pixels in the feature map for each channel. These output feature vectors will be sent straight to the classifier, providing each channel with useful information (Gao et al., 2021).

#### 2.2.4 Depth-wise convolution

The model performs more slowly and uses more memory than many other models when convolutional procedures are utilized extensively. We propose to use depth-wise convolution layers rather than standard convolution ones to solve this issue. Depth-wise convolution is a common technique for reducing the parameters and improving computational and representational efficiency. In this procedure, a distinct filter is applied to each input channel, and a point-wise 1 × 1 convolution is used to merge the outputs.


(2) $$\begin{array}{l} {Ĉ}_{k,l,a}=\underset{i,j}{\sum }{\hat{K}}_{i,j,a}.Z_{k+i-1,l+j-1,a} \end{array}$$

The depth-wise convolution filter of size *P*_*K*_×*P*_*K*_×*X* is denoted by $\hat{K}$ in Equation 2, where *X* is the sum of the input channels and *P*_*K*_ is the spatial dimension. Here, the *ath* channel for the filtered feature map Ĉ is created by using the *ath* kernel from $\hat{L}$ in the *ath*-channel of *Z*.


(3) $$\begin{array}{l} P_{K}\cdot P_{K}\cdot X\cdot P_{Z}\cdot P_{Z} \end{array}$$

(4) $$\begin{array}{l} P_{K}\cdot P_{K}\cdot X\cdot P_{Z}\cdot P_{Z}+X\cdot Y\cdot P_{Z}\cdot P_{Z} \end{array}$$

(5) $$\begin{array}{l} \frac{P_{K}\cdot P_{K}\cdot X\cdot P_{Z}\cdot P_{Z}+X\cdot Y\cdot P_{Z}\cdot P_{Z}}{P_{K}\cdot P_{K}\cdot X\cdot Y\cdot P_{Z}\cdot P_{Z}} \end{array}$$

(6) $$\begin{array}{l} \frac{1}{Y}+\frac{1}{P_{K}^{2}} \end{array}$$

Equation 3 can be used to calculate the depth-wise convolution's computational cost, It is less expensive than the computing cost of the regular convolution operation. *Y* is the total output channels. A 1 × 1 point convolution is used to merge the depth-wise convolutions. The total cost, including the point convolution, can be written as Equation 4. Equations 5, 6 can express the overall cost reduction (Hazarika et al., 2022b,a).

#### 2.2.5 Squeeze and excitation (SE) block

The global features at the channel level are initially extracted by the SE module using the Squeeze function on the feature map. It then presents the Convolution function. The global features are then subjected to an application of the Excitation function in order to ascertain the weights of each channel and comprehend their interrelation. Multiplying the recently added mapping feature yields the final features.

To build a Squeeze-and-Excitation block, begin with a convolution operation *F*_*tr*_ translating an input X ∈ ℝ*H*^′^ × *W*^′^ × *C*^′^ to feature mappings U ∈ ℝ^*H*×*W*×*C*^. We use a collection of learned kernels, *V* = [*v*_1_, *v*_2_, ....*v*_*C*_], where *v*_*C*_ denotes the parameters of the *Cth* filter. Show the outputs as *U* = [*u*_1_, *u*_2_, ....*u*_*C*_],


(7) $$\begin{array}{l} u_{c}=v_{c}*X=\overset{C^{\prime }}{\underset{s=1}{\sum }}v_{c}^{s}*x^{s} \end{array}$$

where “*" is the convolution operation and $v_{c}^{s}$ is a 2D spatial kernel that translates to a single channel of *v*_*c*_ on the corresponding channel of *X*. Channel dependencies are implicitly incorporated in *v*_*C*_ as the output is the total of all channels. The filters capture the local spatial correlation (Hu et al., 2018; Yang et al., 2022).

The model's attention mechanism allows it to suppress the less important channel properties and prioritize the more informative ones. This attention mechanism memorizes the spatial correlations between the information after receiving the spatial properties in a channel as input. Under the convolution results of each channel, the sum of the channels has been calculated concurrently, and the spatial relations learned by the convolution kernel can be merged with the relationships between channel characteristics. To fully describe channel-wise dependencies, it has to learn a nonlinear interaction between channels and a mutually exclusive relationship. To meet these needs, we employed a straightforward gating mechanism with Sigmoid activation.


(8) $$\begin{array}{l} z_{c}=F_{sq}\left(u_{c}\right)=\frac{1}{H\times W}\overset{H}{\underset{i=1}{\sum }}\overset{W}{\underset{j=1}{\sum }}u_{c}\left(i,j\right),z\in {\mathbb{R}}^{C} \end{array}$$

(9) $$\begin{array}{l} s=F_{ex}\left(z,W\right)=\sigma \left(g\left(z,W\right)\right)=\sigma \left(W_{2}\delta \left(W_{1}z\right)\right) \end{array}$$

*W* and *H* are the width and height of the mapping features, respectively and $W_{1}\in {\mathbb{R}}^{\frac{C}{r}\times C}$, $W_{2}\in {\mathbb{R}}^{C\times \frac{C}{r}}$

To simplify the model and enhance its capacity for generalization, two fully connected (FC) design layers have been employed. The first FC layer contributes to dimension reduction, and “*r*" is a parameter for the dimension reduction factor. ReLU activation can then be performed. The last layer of FC adds a new dimension. New features on *U* can eventually be multiplied by the learning activation levels of each channel. The final output of the block is derived by rescaling *U* using the activations ‘*s*":


(10) $$\begin{array}{l} \tilde{x_{c}}=Fscale\left(u_{c},s_{c}\right)=s_{c}u_{c} \end{array}$$

where $\tilde{X}=\left[\tilde{x_{1}},\tilde{x_{2}},....\tilde{x_{C}}\right]$ and *Fscale*(*u*_*c*_, *s*_*c*_) denote the channel-wise multiplication between the scalar *s*_*c*_ and the feature map ${\text{u}}_{\text{c}}\in {\mathbb{R}}^{H\times W}$. Equation 7 through Equation 10 provide a mathematical representation of the full function (Hu et al., 2018; Gao et al., 2021; Alazwari et al., 2024).

#### 2.2.6 Deep CNN_ResNet-18 based scratch model with SE and depth wise convolution

**Figure 6:**
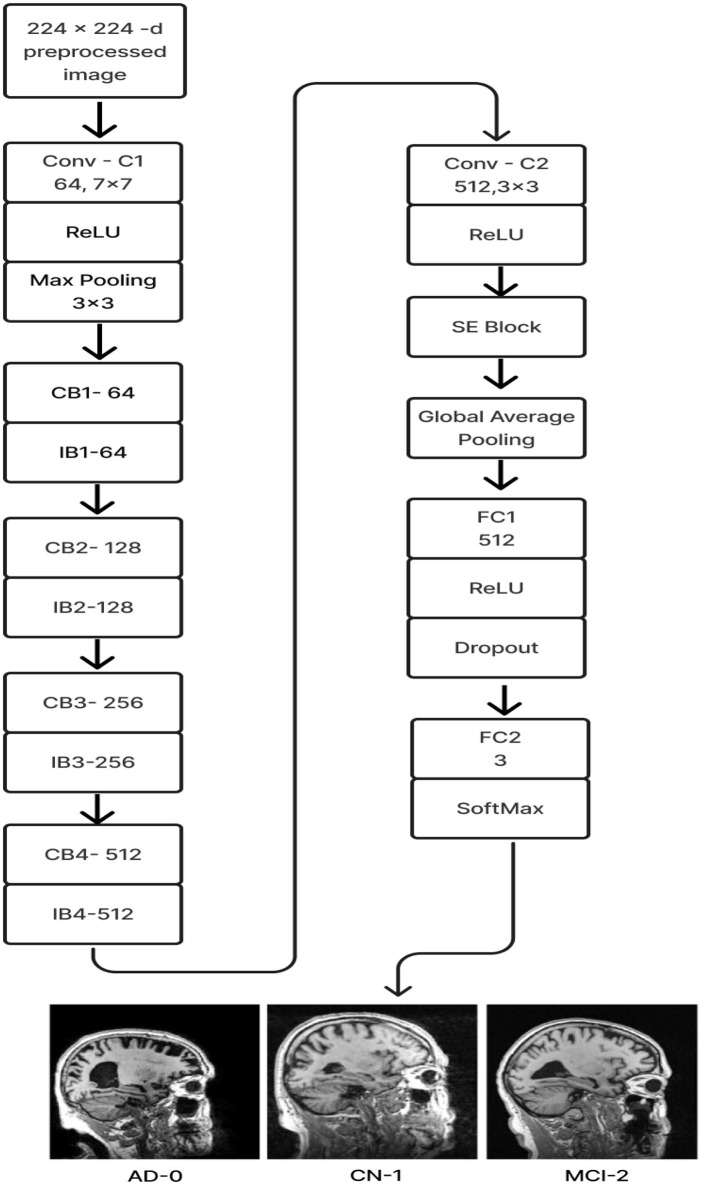
Proposed model 1: deep CNN_ResNet-18 based scratch model with SE and depth convolution.

**Figure 7:**
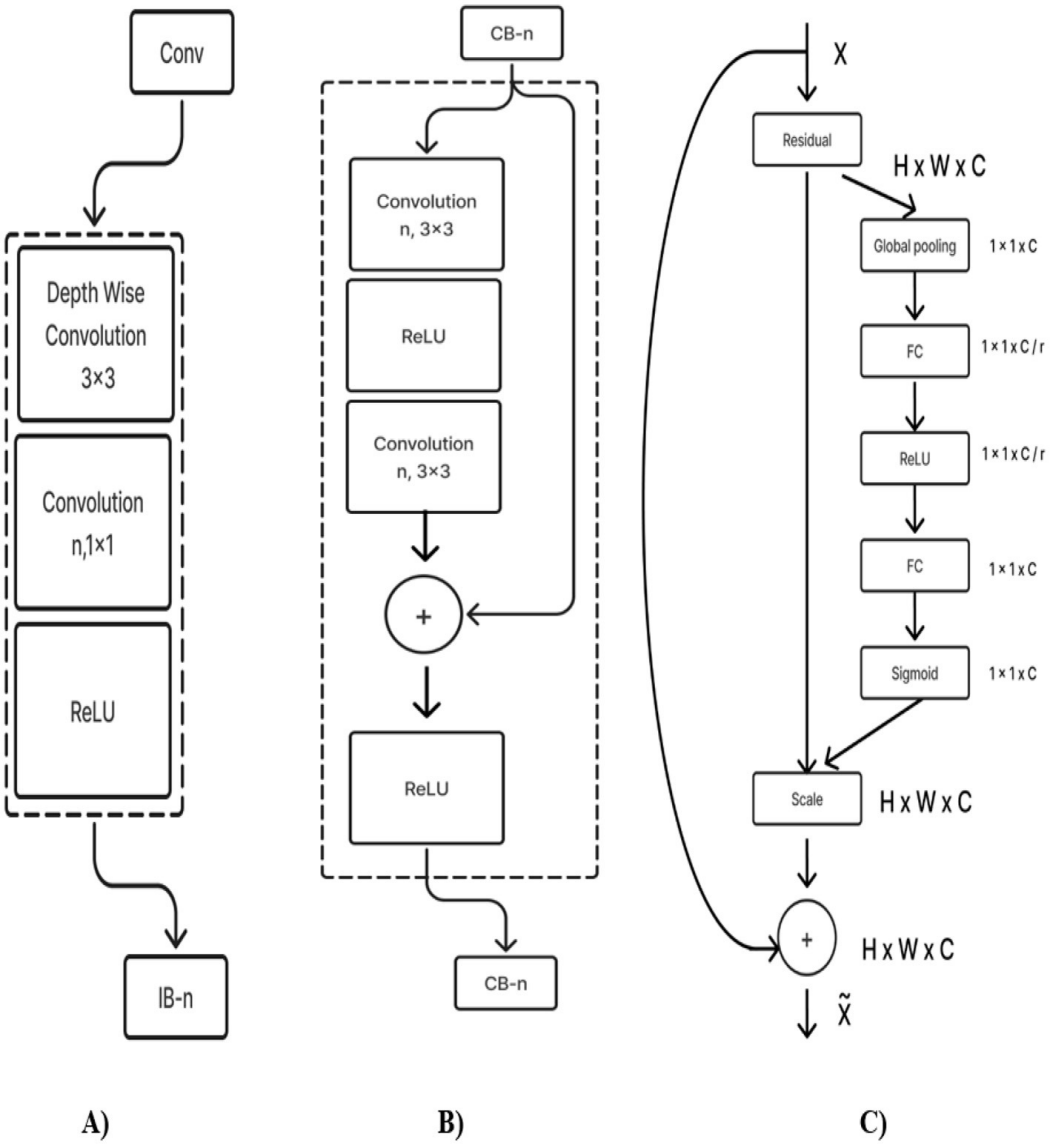
**(A)** Convolution block (CB). **(B)** Identity block (IB). **(C)** squeeze and excitation block (SE).

The proposed Deep CNN model was built on the following principles: Squeeze and Excitation (attention mechanism), Depth convolution, and Residual structure. The proposed architecture is shown in Figure 6. We see the Convolution Block (CB-Block), Identity Block (IB-Block), and Squeeze and Excitation Block (SE-Block) in Figure 7. The following describes this architecture's key components. A Squeeze and Excitation (SE) block is integrated with a Deep CNN model that is based on a ResNet-18 model and was created from scratch using a unique architecture. Moreover, Depth convolution was used instead of regular convolution. The Convolution blocks, Identity blocks, and SE blocks are used in this six-part architecture. This proposed model with SE block consists of two convolution layers (C1 and C2), four convolution blocks (CB1, CB2, CB3, and CB4), four identity blocks (IB1, IB2, IB3, and IB4), SE Block, Global Average Pooling layer, and two Fully Connected layers (FC1 and FC2).

Batches of sagittal slices from MRI brain scans with dimensions of 224 × 224 × 1 are accepted as input. A max-pooling layer with a kernel size of 3 × 3, a batch normalization layer, a ReLU activation function, and a convolutional layer with a kernel size of 7 × 7 are employed in the first section, C1. The max-pooling layer preserves important feature information while reducing the model's size and parameters and expanding the receptive fields. Beginning with a depth-wise convolution, the Convolution Block in the second section proceeds with a convolution layer and ReLU activation. Afterward, an Identity Block, which comprises the ResNet-18 model's residual portion, is employed to address the vanishing and exploding gradient issues that most deep-learning networks encounter. The third part, which consists of convolution C2 followed by ReLU activation, receives the combined output of the second part as input. The fourth part, SE-Block, includes the Squeeze-and-Excitation (SE) module.

The SE module has two main components. Squeeze is the first method, causing the input image to undergo Global Average Pooling and compressing the feature map into a 1 × 1 × C vector. Excitation is the second, consisting of two densely connected layers and two activation functions (Sigmoid and ReLU). The first fully connected layer receives 1 × 1 × C as input and outputs 1 × 1 × C × 1/*r*, where “*r*" is a reduction parameter that lowers the number of channels to reduce computation. The second fully connected layer receives 1 × 1 × C × 1/*r* as input, and 1 × 1 × C is the output. This study uses *r* = 16. After obtaining the output vector, the initial feature map and the 1 × 1 × C vector will be scaled. The initial feature map measures W × H × C. The final output result is obtained by multiplying the weight value of each channel output by the SE module by the two-dimensional matrix of the corresponding channel of the initial feature map. The output size of this layer was set to 1 × 1 in the fifth section, which used Global Average Pooling, ReLU, and Dropout. The sixth section uses a fully connected classification Layer with three neurons in the output related to predicting three Alzheimer's stages. Features ranging from low to high could be extracted by the modification of ResNet-18. The model's sensitivity to channel features is increased by recalibrating the starting features in the channel dimension. This allows the model to identify the salient aspects of different channels automatically. Lowering the training parameters, accelerating the model's rate of convergence, and improving classification accuracy are all accomplished via the Global Average Pooling layer. By using depth convolution and the SE block, we could reduce the training parameters from 11.189 to 9.125 million. This resulted in training the model taking 1,915.59 s, testing taking 1 s, and real-time evaluation taking 17 ms.

### 2.3 ResNet-18 pre-trained model with SE

**Figure 8:**
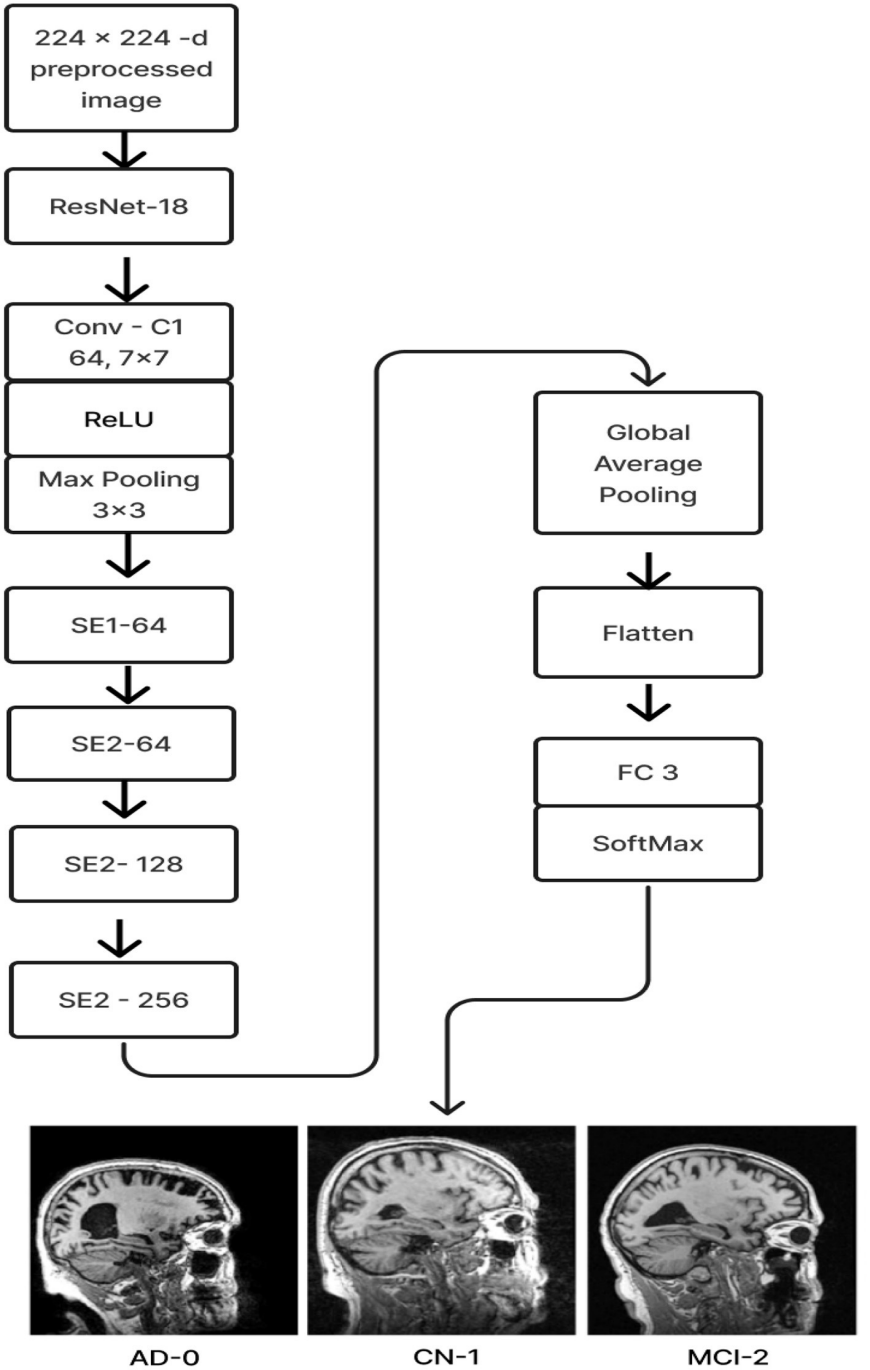
Proposed model 2: ResNet-18 pre-trained model with SE block.

In this model, we employ transfer learning to optimize the training of gathered data and avoid overfitting issues. Figure 8 displays the above-prescribed model.

The ResNet-18 pretraining model initializes the weights (ImageNet) of 17 convolutional layers, with the exception of SE-Block (Gao et al., 2021), during the training phase. Once the learned weights were loaded, the entire model was retrained using the current ADNI dataset. This can increase training speed and accuracy and enhance the model's ability to detect illness stages from the existing MRI image collection. For feature learning to be steady and successful, this is essential. The Squeeze and Excitation components make up the ResNet-18 pre-trained model with SE. Every SE block adaptively re-calibrates channel-wise feature responses by simulating channel interdependencies and embedding the knowledge globally. ResNet-18, the pre-training model, was used as the first layer, and the last fully connected layer of the ResNet-18 model was replaced by four SE blocks integrated as the next four layers. After that, the features were flattened and averaged. Ultimately, a fully connected layer made up of three neurons with a softmax activation function is linked in order to categorize the three stages of Alzheimer's disease.

### 2.4 ResNet-18 pre-trained model without SE

**Figure 9:**
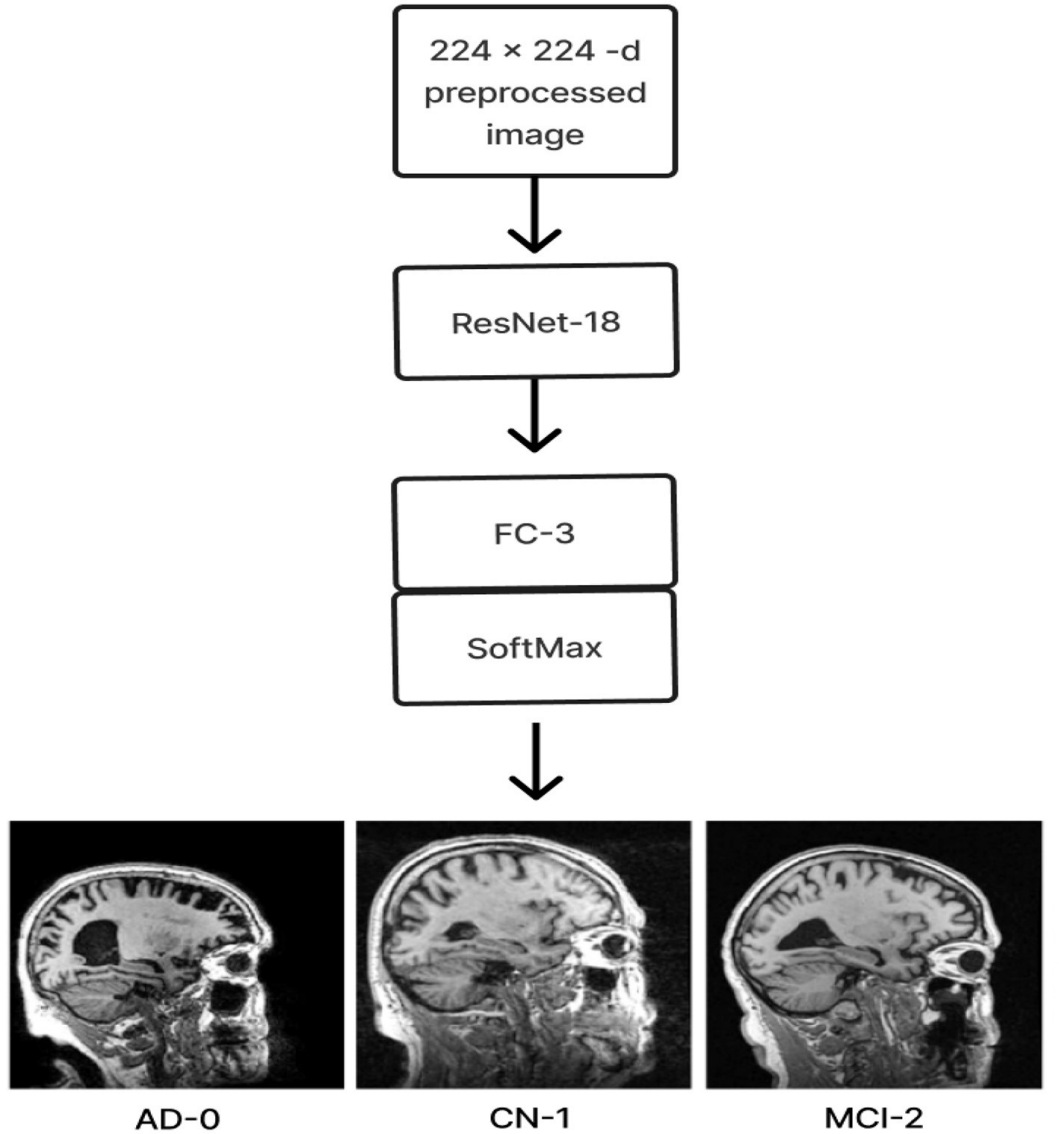
Model 3: ResNet-18 pre-trained model without SE block.

The ResNet-18 pre-training model (freezing all layers except the last) is the first layer of the ResNet-18 pre-training model without SE. To identify three distinct stages of Alzheimer's disease, a classification layer consisting of three neurons with softmax activation functions follows. Using the existing ADNI dataset, the model was retrained to take advantage of the transfer learning principle and improve performance after loading the learned weights from ImageNet. Figure 9 displays the architecture for the same.

### 2.5 Experimental setup

For all the experimental investigations, we used a Dell PowerEdge R760XA Server GPU node, an Intel Xeon Gold 6438Y+, 32-core, 2 GHz processor, 512 GB of RAM, DDR5 4800MT/s, 1.92 TB of SSD, and an X Nvidia Hopper H100 (80 GB) with Windows 11.

## 3 Experiments results and discussion

This section analyzes the performance of three models with regard to various hyperparameters in terms of the confusion matrix, ROC values, and training and test accuracy. Analyses of prediction accuracy and convergence on a few criteria were also carried out. Using a new dataset called OASIS-1 and real-time patient MRI scans, along with their demographic data, also showed how all models were evaluated.

### 3.1 Comparisons of three model performance

**Figure 10:**
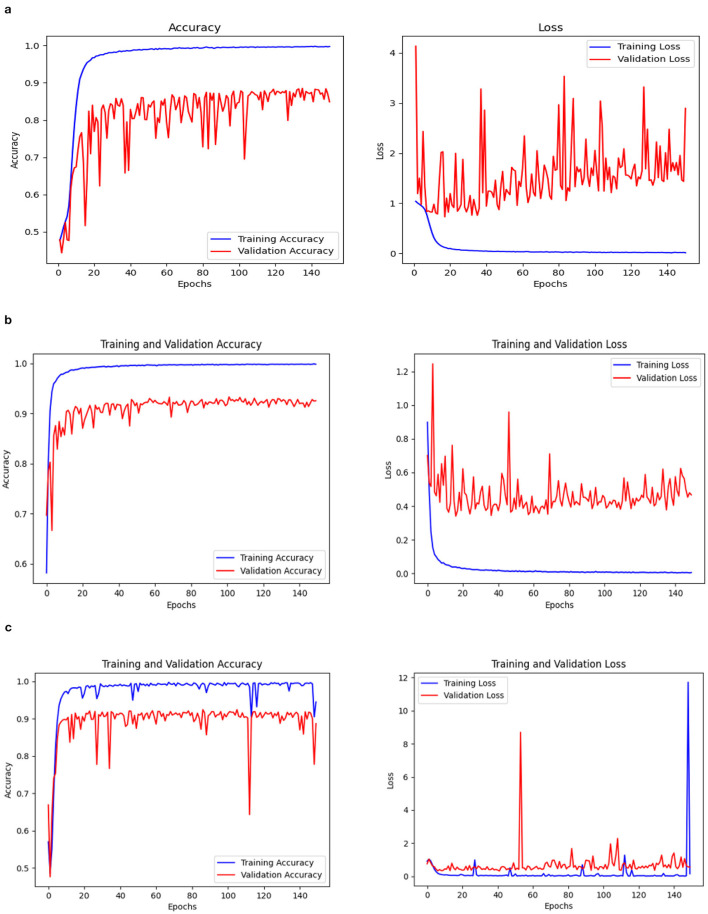
Accuracy and loss curve. **(A)** Model 1. **(B)** Model 2. **(C)** Model 3.

Three models, (1) DeepCNN ResNet-18 based scratch model with SE, (2) ResNet-18 pre-trained model with SE, and (3) ResNet-18 pre-trained model without SE, have classification best test accuracies of 88.51% (135*th* epoch), 93.26% (69*th* epoch), and 92.41% (106*th* epoch), respectively. The three models displayed nearly identical best training accuracies with 99.81% (142^*nd*^ epoch), 99.93% (149*th* epoch), and 99.73% (68*th* epoch). Figure 10 depicts all three models' accuracy and loss curves.

The ROC values were the major criterion for evaluating these models because the ADNI dataset is highly imbalanced, even after augmentation techniques. The ROC values for the three models were AD:95, CN:95, MCI:93 for Model 1; AD:98, CN:99, MCI:98 for Model 2; and AD:94, CN:97, MCI:94 for Model 3. These values demonstrate the efficacy of identifying the various phases of Alzheimer's disease.

**Figure 11:**
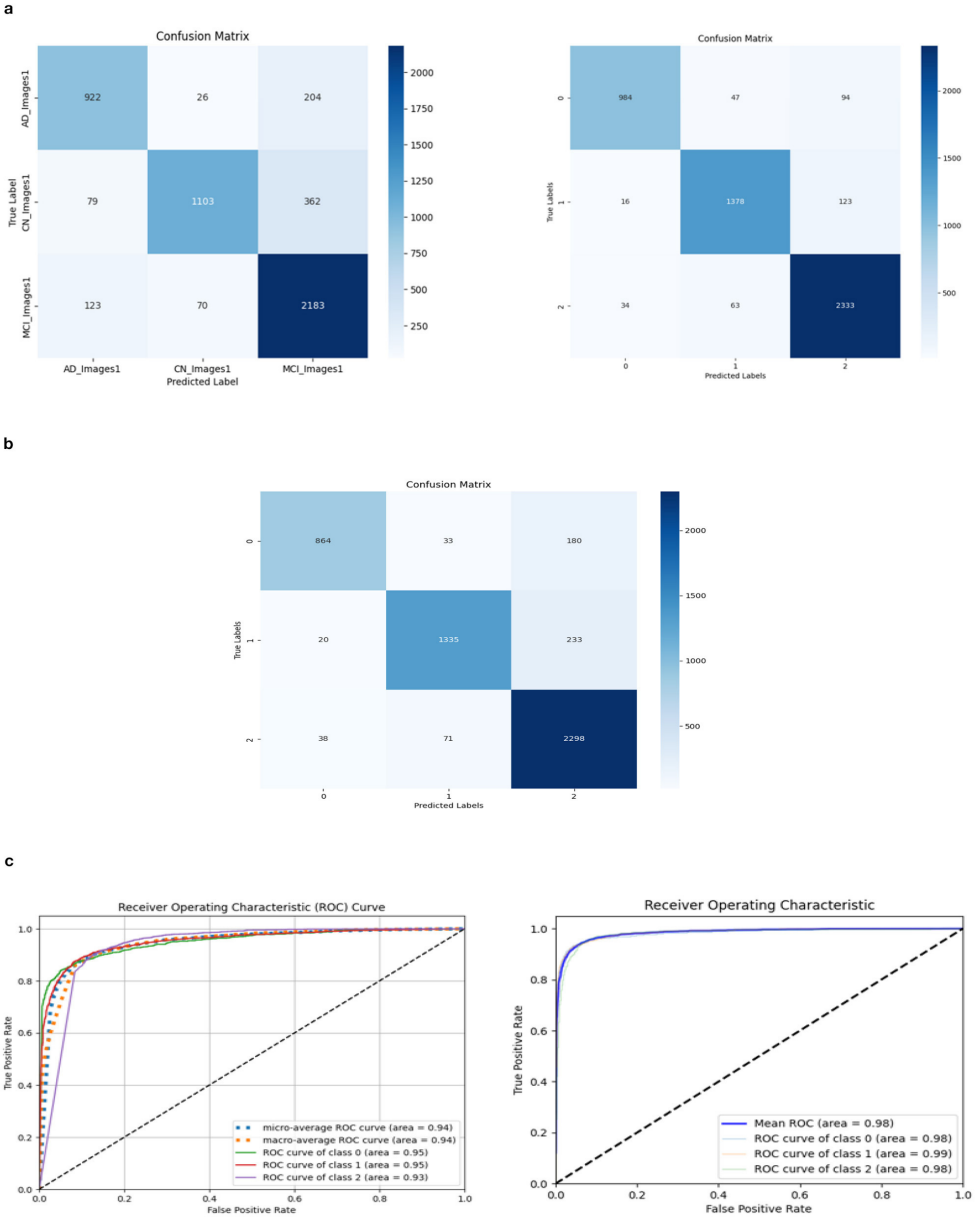
Confusion matrix and ROC. **(A)** Confusion matrix of Model 1 and 2. **(B)** Confusion matrix of Model 3. **(C)** ROC curve of Model 1 and 2.

**Figure 12:**
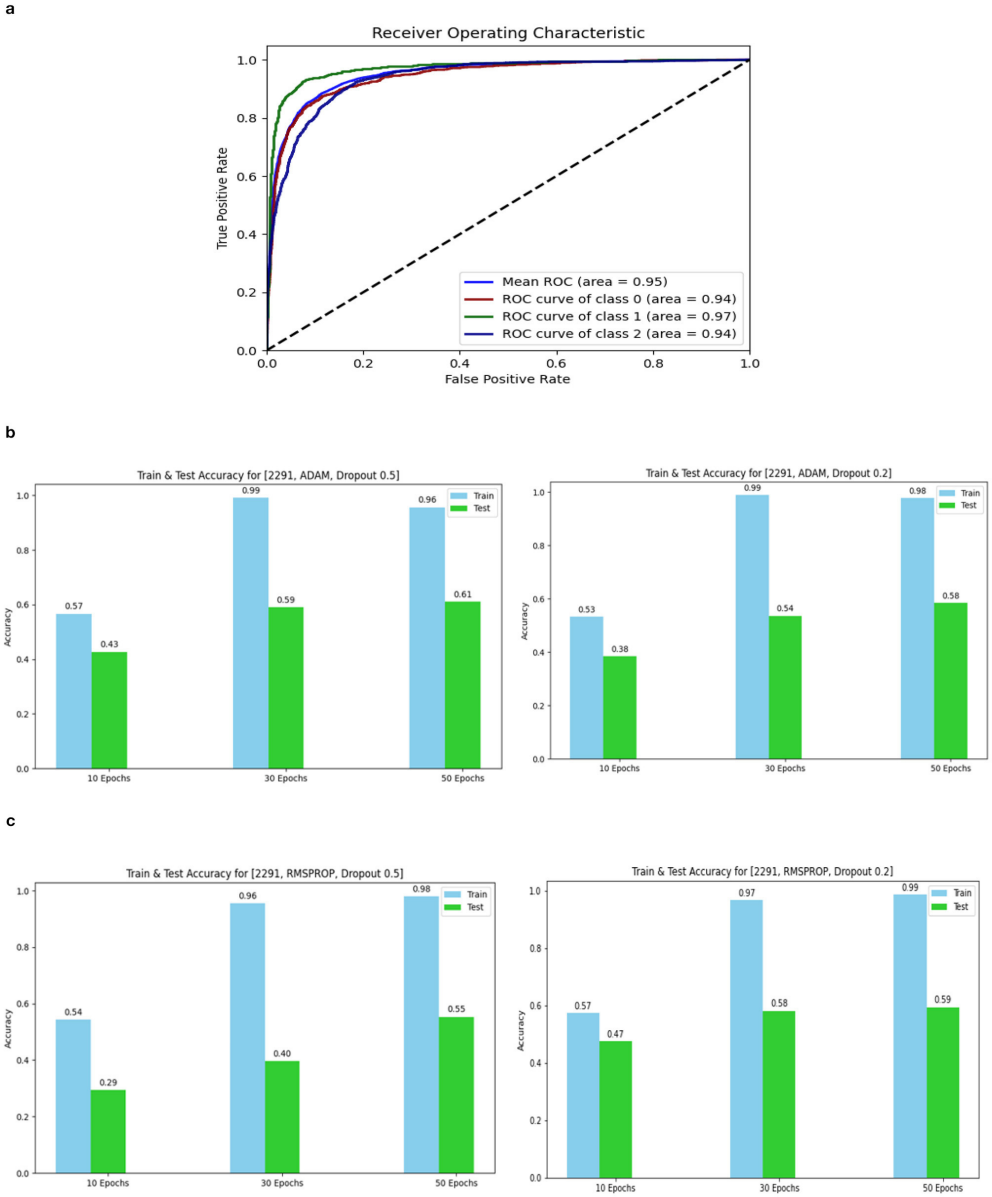
ROC curve of Model 3 and Accuracy for 2,291 data 1. **(A)** ROC curve of Model 3. **(B)** 2,291 and Adam and Dropout. **(C)** 2,291 and RMSProp and Dropout.

**Figure 13:**
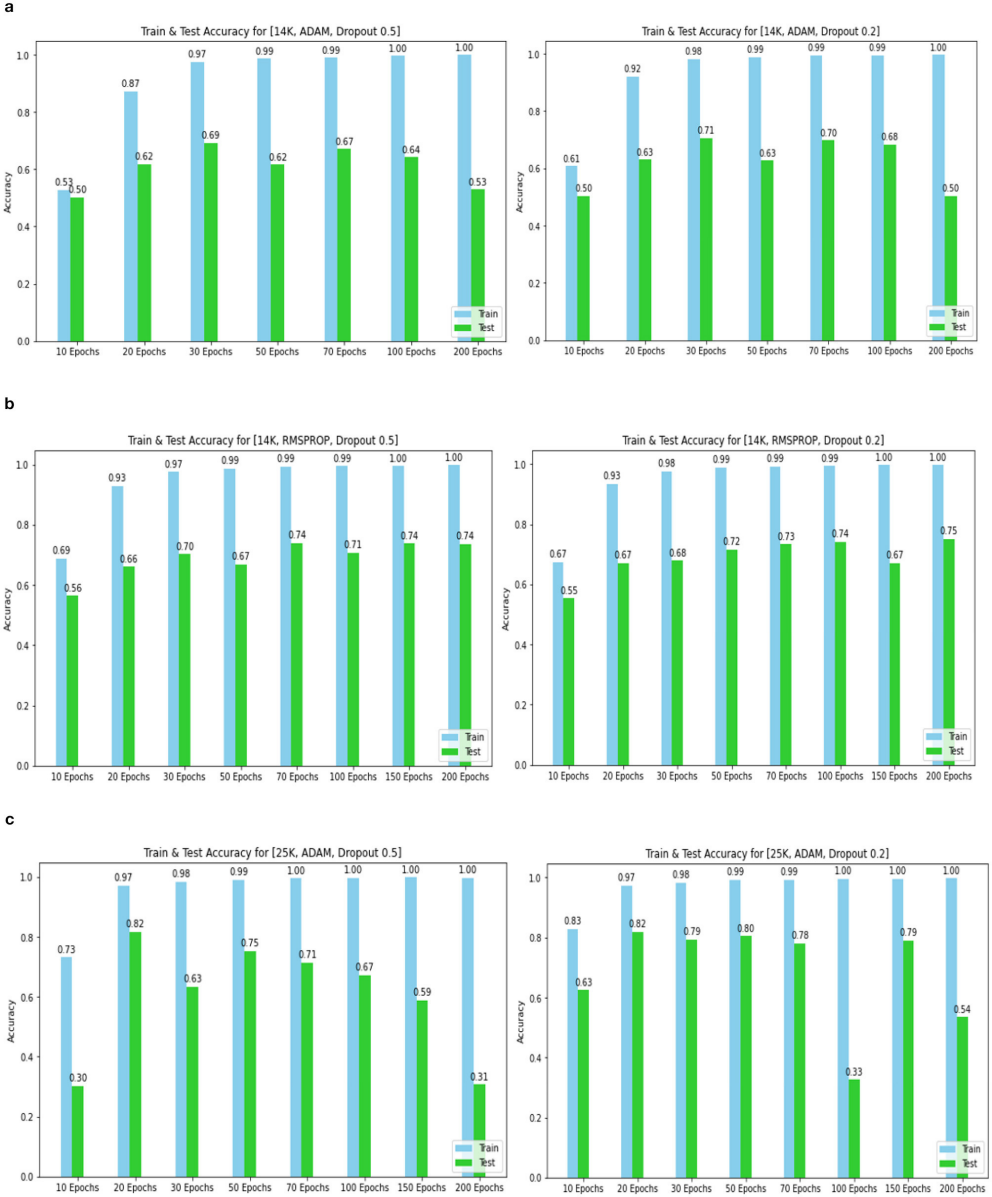
Training and test accuracy for 14k and 25k data and Optimizer and Dropout. **(A)** 14k data and Adam and Dropout. **(B)** 14k data and RMSProp and Dropout. **(C)** 25k data and Adam and Dropout.

**Figure 14:**
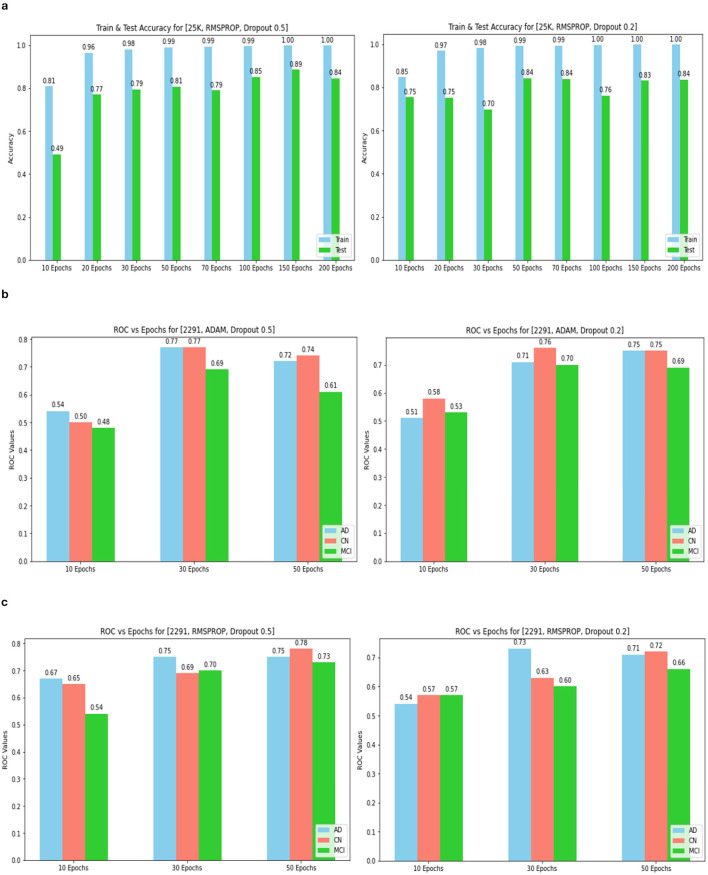
Training and tEST aCCURacy for 25k data and ROC for 2,291 and Optimizer and Dropout. **(A)** 25k data and RMSProp and Dropout. **(B)** 2,219 data and Adam and Dropout. **(C)** 2,219 data and RMSProp and Dropout.

**Figure 15:**
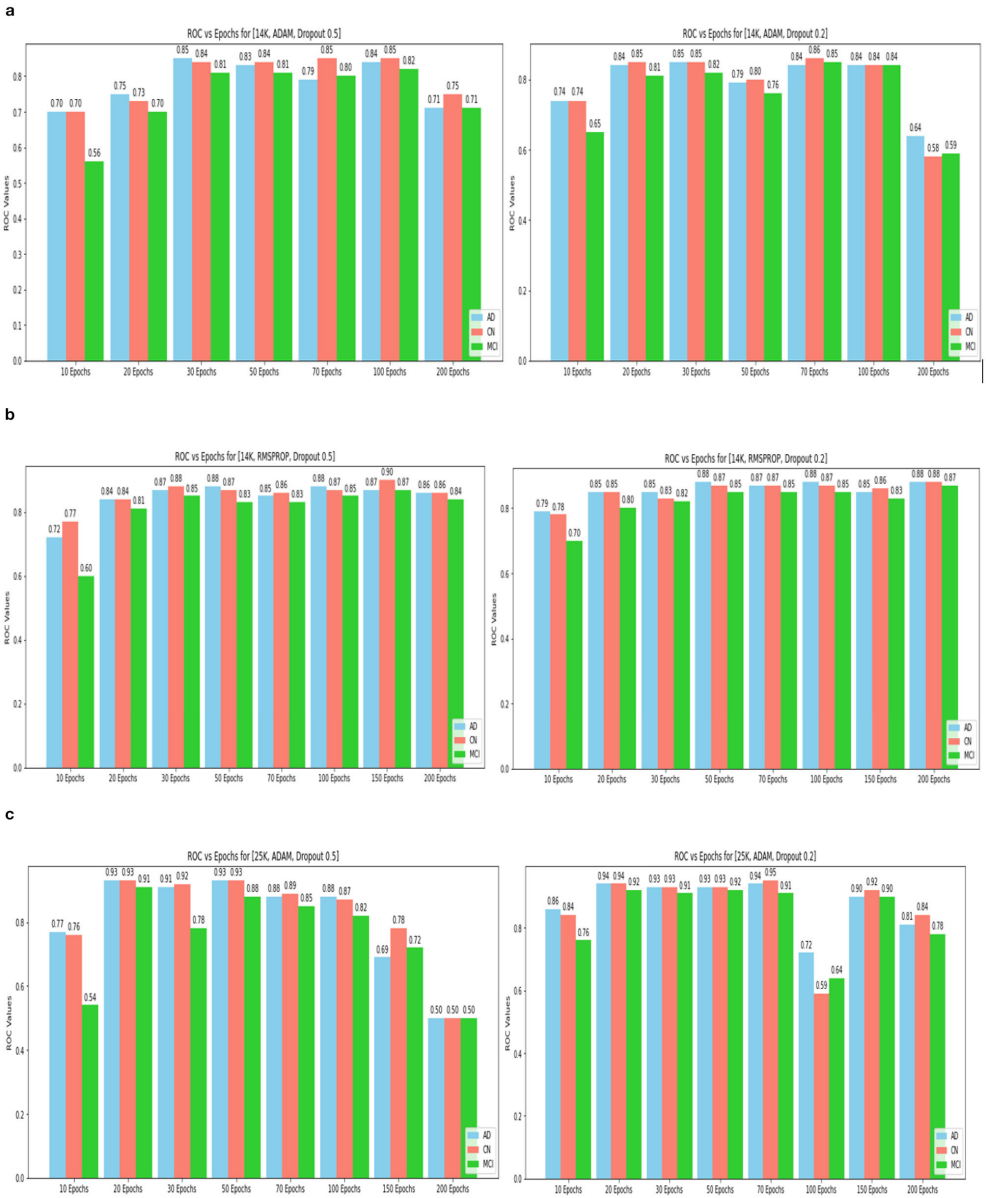
ROC values(AD, CN, MCI) for 14k and 25k data and Optimizer and Dropout. **(A)** 14k data and ADAM and Dropout. **(B)** 14k data and RMSProp and Dropout. **(C)** ROC values (AD, CN, MCI) for 25k data and ADAM and Dropout.

**Figure 16:**
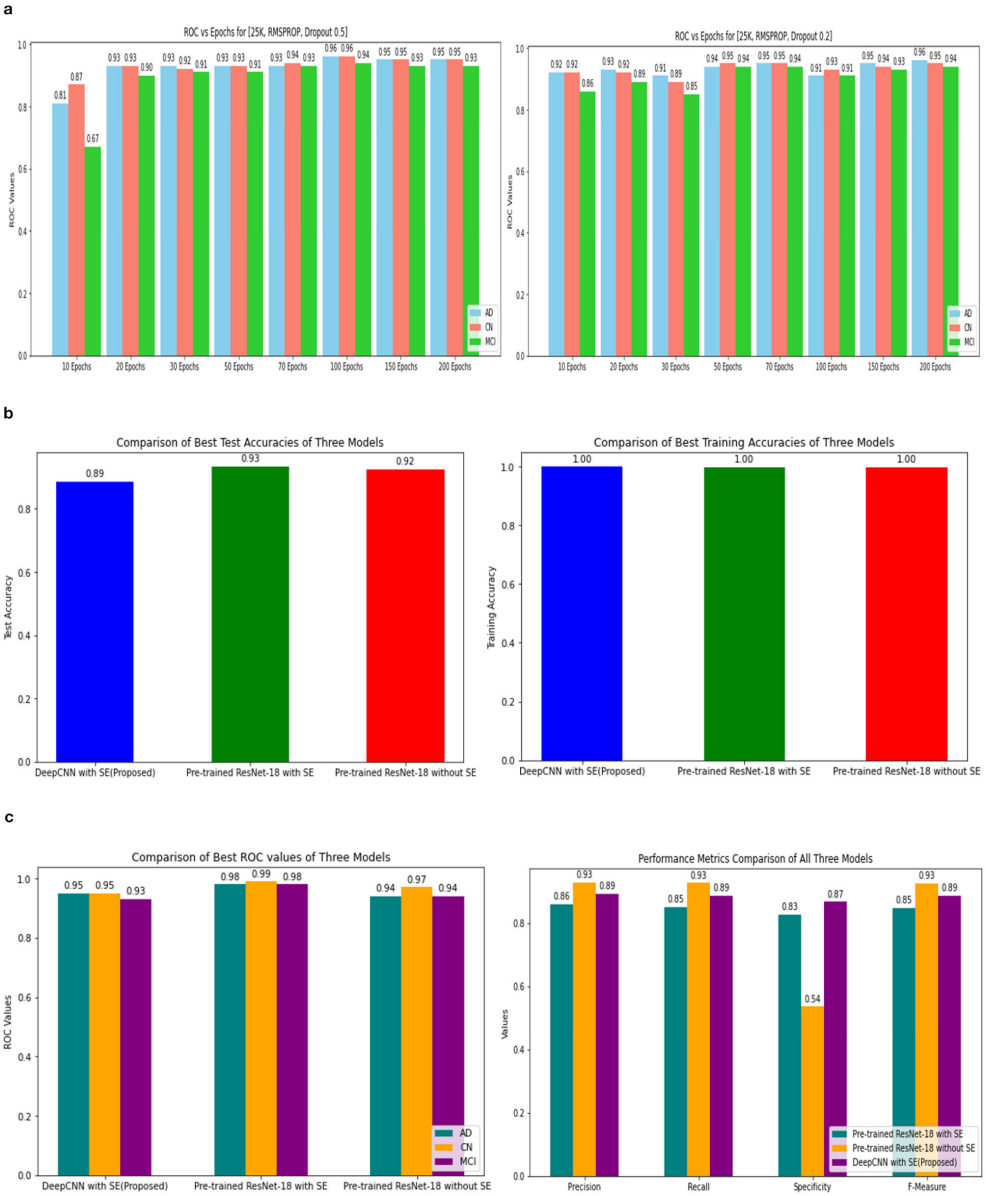
ROC values of 25k data and Metrics values comparison. **(A)** 25k data and RMSProp and Dropout. **(B)** Testing and training accuracy comparison of 3 Models. **(C)** Metrics values comparison of 3 Models.

The confusion matrix and ROC values for each of the three models are shown in Figure 11. Several regularization strategies, including adding a dropout layer, increasing epochs, altering the optimizer, and providing data augmentation to the original dataset prior to training, have been used to lower generalization errors or avoid overfitting. It was discovered that all of the strategies significantly improved the accuracy and ROC values. A better representation of ROC values and improvisation of testing and training accuracies is displayed from Figures 12–15. In order to generalize the model and lessen overfitting, cross-validation procedures were also employed in an 80:20 ratio on the original dataset. The comparison of test and training accuracies, ROC values, and other evaluation metrics for each of the three models are interpreted in Figures 16B, C.

The pre-trained ResNet-18 model with SE Blocks produced the best results for every comparison because of the efficiency of using pre-trained models and the decrease in the number of training parameters. The network that is trained from scratch using deep learning techniques, unfortunately, has the following drawbacks: (a) It requires a large amount of labeled training data, which could be problematic for the medical field where doctors annotate the data (b) It can be expensive; and (c) It requires a significant amount of computational resources. (c) These models also require a lot of hyper-parameters to be carefully and laboriously adjusted, which can result in either over-fitting or under-fitting and, ultimately, poorer performance. (d) Using a tiny set of medical training data can trap the cost function in a local minima problem. Test accuracy, training accuracy, and ROC values were examined with respect to several data augmentation levels, epochs, optimizer, and dropout stages. Convergence accelerated with increasing data quantity and epoch. Overall, the optimizer RMSprop generated speedy convergence. On the 14K dataset, Dropout 0.2 outperformed, whereas on the 25K dataset, Dropout 0.5 produced superior outcomes. Test accuracy and ROC values were the primary metrics used to assess the model's effectiveness. The test and training accuracy during hyperparameter tuning are provided in Tables 1–3. The Confusion Matrix evaluates how well the classification model predicts a category label for each input dataset. Accuracy is the ratio of accurately anticipated cases to all other cases. The precision of each category is used to calculate the proportion of all predicted positive instances. Recall shows the proportion of all actual positive cases among anticipated positive occurrences.

**Table 1:** Test and training accuracy, ROC values: 2,291 (original) dataset vs. Epoch vs. Optimizer vs. Dropout.

**Dataset**	**Epoch**	**Optimizer**	**Dropout-0.5**		**ROC**			**Dropout-0.2**		**ROC**		
		Adam	Training	Testing	AD	CN	MCI	Training	Testing	AD	CN	MCI
2,291	10		56.67	42.7	54	50	48	53.24	38.34	51	58	53
	30		**99.2**	**59.04**	**77**	**77**	**69**	98.9	53.59	71	76	70
	50		95.63	61	72	74	61	97.74	58.38	75	75	69
		RMSprop	Training	Testing	AD	CN	MCI	Training	Testing	AD	CN	MCI
	10		54.32	29.41	67	65	54	57.29	47.49	54	57	57
	30		95.58	39.65	75	69	70	96.59	58.17	73	63	60
	50		98.04	55.33	**75**	**78**	**73**	**98.59**	**59.26**	71	72	66

**Table 2:** Test and training accuracy, ROC values: 14K Dataset vs. Epoch vs. Optimizer vs. Dropout.

**Dataset**	**Epoch**	**Optimizer**	**Dropout (0.5)**		**ROC**			**Dropout (0.2)**		**ROC**		
		Adam	Training	Testing	AD	CN	MCI	Training	Testing	AD	CN	MCI
14k	10		52.73	50.16	70	70	56	60.84	50.37	74	74	65
	20		87.3	61.72	75	73	70	92.14	63.04	84	85	81
	30		97.5	69.24	85	84	81	98.13	70.66	85	85	82
	50		98.64	61.65	83	84	81	98.75	62.7	79	80	76
	70		98.89	67.17	79	85	80	99.41	69.83	84	86	85
	100		99.6	64.23	84	85	82	99.47	68.39	84	84	84
	200		99.96	52.95	71	75	71	99.64	50.37	64	58	59
		RMSprop	Training	Testing	AD	CN	MCI	Training	Testing	AD	CN	MCI
	10		68.69	56.4	72	77	60	67.44	55.42	79	78	70
	20		92.86	66.02	84	84	81	93.44	67.17	85	85	80
	30		97.5	70.22	87	88	85	97.52	67.95	85	83	82
	50		98.64	66.77	88	87	83	98.91	71.65	88	87	85
	70		99.28	73.98	85	86	83	99.16	73.34	87	87	85
	100		99.41	70.7	88	87	85	99.41	74.22	88	87	85
	150		99.53	73.78	87	90	87	99.69	67.01	85	86	83
	200		99.82	73.51	86	86	84	99.7	75.03	88	88	87

**Table 3:** Test and training accuracy, ROC values: 25K Dataset vs. Epoch vs. Optimizer vs. Dropout.

**Dataset**	**Epoch**	**Optimizer**	**Dropout (0.5)**		**ROC**			**Dropout (0.2)**		**ROC**		
		Adam	Training	Testing	AD	CN	MCI	Training	Testing	AD	CN	MCI
25k	10		73.16	30.26	77	76	54	82.96	62.52	86	84	76
	20		97.18	81.62	93	93	91	97.37	81.98	94	94	92
	30		98.4	63.29	91	92	78	98.24	79.42	93	93	91
	50		99.09	75.2	93	93	88	99.19	80.4	93	93	92
	70		99.57	71.41	88	89	85	99.27	78.08	94	95	91
	100		99.68	67.29	88	87	82	99.64	32.57	72	59	64
	150		99.89	58.77	69	78	72	99.6	79.04	90	92	90
	200		99.73	30.776	50	50	50	99.77	53.53	81	84	78
		RMSprop	Training	Testing	AD	CN	MCI	Training	Testing	AD	CN	MCI
	10		80.86	49.15	81	87	67	84.7	75.37	92	92	86
	20		96.38	76.91	93	93	90	96.96	75.24	93	92	89
	30		97.95	79.46	93	92	91	98.26	69.72	91	89	85
	50		99.01	80.68	93	93	91	99.14	84.29	94	95	94
	70		99.33	78.9	93	94	93	99.42	83.97	95	95	94
	100		99.46	85.23	96	96	94	99.64	76.2	91	93	91
	150		99.81	88.51	95	95	93	99.81	83.04	95	94	93
	200		99.76	84.46	95	95	93	99.85	83.66	96	95	94

The Confusion Matrix is used to compute the Precision, Recall, Specificity, and F1 score in order to assess each model's performance. The metric values for each of the three models are shown in Table 4. The second model, which uses the Transfer Learning and Squeeze and Excitation principles, is the best at detecting three AD biomarkers, as Table 4 shows. By adding appropriate parameters, such as integrating the SE block and employing Depth-wise convolution instead of the traditional one to reduce tuning parameters, we have improved the efficiency of the suggested Deep CNN ResNet-18 model. In the same field, state-of-the-art techniques that are either developed from scratch using SE blocks or that use pre-training models with or without SE blocks are listed in Table 5. All of our approaches demonstrated a faster rate of convergence than earlier scholars' attempts, and Table 6 compares the values of all significant evaluation metrics of their work to the suggested one.

**Table 4:** Metrics values.

	**Model 1**	**Model 2**	**Model 3**
Precision	85.83	92.64	89.17
Recall	84.90	92.57	88.66
Specificity	82.68	53.51	86.59
F-Measure	84.68	92.54	88.59

**Table 5:** State_of_art_Methods.

**References**	**Dataset**	**Classification**	**Accuracy**	**Model**
	**Scratch model**			
Odusami et al. (2021)	ADNI	AD vs. CN	75.12	ResNet-18(scratch)
Lu et al. (2022)	ADNI	MCI vs. AD	70.7	3D ResNet-18 (scratch)
Dharwada et al. (2024)	ADNI	AD vs. CN	87.37	3D ResNet-50 +SE(scratch)
Francis et al. (2024)	ADNI	MCIn vs. MCInc	82	Local Binary Pattern +Conv
**Proposed**	**ADNI**	**AD vs. CN vs. MCI**	**88.51**	**Deep CNN ResNet-18 based** **+ SE**
	**Pre-trained Model +SE**			
Illakiya et al. (2023)	ADNI	AD vs. CN	89.17	Dense-169+SE
Dharwada et al. (2024)	ADNI	AD vs. CN	89.25	3D ResNet-50 +SE
Zhang et al. (2024)	ADNI	AD vs. CN	84.08	VGG-16+SE
**Proposed**	**ADNI**	**AD vs. CN vs. MCI**	**93.26**	**Pre-trained ResNet-18** **+ SE**
	**Pre-trained model without SE**			
Illakiya et al. (2023)	ADNI	AD vs. CN	77	Dense-169
Tajammal et al. (2023)	ADNI	AD vs. CN	87.5	ResNet-18
Suja and Raajan (2024)	ADNI	AD vs. CN	80.19	ResNet-18
Zhang et al. (2024)	ADNI	AD vs. CN	82.94	VGG-16
Suja and Raajan (2024)	ADNI	AD vs. CN	80.19	VGG-16
Topsakal and Lenkala (2024)	ADNI	AD vs. CN	74.9	ResNet-50
**Proposed**	**ADNI**	**AD vs. CN vs. MCI**	**92.41**	**Pre-trained ResNet-18** **without SE**

**Table 6:** Metrics values comparison of State_of_art_Methods.

**References**	**Precision**	**Recall**	**Specificity**	**F1-score**	**Accuracy**
**Scratch model**					
Odusami et al. (2021)		**97.8**	50.59		75.12
Lu et al. (2022)		67.9	73.3	69.1	70.7
Dharwada et al. (2024)	**88.09**	84.22	**87.87**	84.52	87.37
Francis et al. (2024)	82	82	82	82	82
**Proposed**	85.83	88.51	82.68	**84.68**	**88.51**
**Pre-trained model + SE**					
Illakiya et al. (2023)	**96**	89	92		89.17
Dharwada et al. (2024)	88.09	84.22	**87.87**	84.52	89.25
Zhang et al. (2024)		80.63	86.32		84.08
**Proposed**	92.64	**92.57**	53.51	**92.54**	**93.26**
**Pre-trained model without SE**					
Illakiya et al. (2023)	75.81	76.37		75.66	77
Tajammal et al. (2023)	85.4	88.6	84.3	86.5	87.5
Suja and Raajan (2024)	84.85	**95.38**	39.01	**89.81**	80.19
Zhang et al. (2024)		81.52	84.81		82.94
Suja and Raajan (2024)	83.95	92.53	29.25	88.04	80.19
Topsakal and Lenkala (2024)					74.9
**Proposed**	**89.17**	88.66	**86.59**	88.59	**92.41**

Bold values indicate the best monitored values for different metrics for different models.

### 3.2 Convergence and prediction accuracy analysis

Convergence and prediction accuracy analysis under several criteria, including Transfer learning, Data Augmentation, Optimization and Dropout, and Adding more SE blocks, are the main topics of this section.

#### 3.2.1 Transfer learning

Pre-trained models use large amounts of data and knowledge transfer techniques to reduce the number of training samples required to complete a task. Compared to training using randomly initialized models, this approach improves accuracy, saves time, and facilitates efficient discriminating across the dataset's many classes. The experiment's findings show that, on the test dataset, the model ResNet-18 with SE's accuracy is 4.75% higher, and the other model, ResNet-18, without SE's accuracy, is 3.9% higher than the model without transfer learning and designed from scratch. Therefore, transfer learning is a better option for having better convergence and prediction accuracy.

#### 3.2.2 Data augmentation

The Deep CNN scratch model's convergence and recognition accuracy values, both with and without data augmentation, are listed in Tables 1–3. The model was trained on different augmentation phases using the same experimental setup, and the final classification accuracy for the first and second augmentation stages in the testing dataset was 73.78% and 88.51%, respectively. Before data augmentation, accuracy was 59.26%; The above tables illustrate that accuracy increased by about 29.25% after augmentation. Figures 12–16, every enhancing effect brought about by augmentation has been shown.

#### 3.2.3 Comparison of optimization algorithms and dropout

To anticipate the best outcome on the final classification, a dropout of 0.5 or 0.2 is used to the custom layer over a fully connected layer in order to prevent overfitting, generalize the data, and minimize validation loss. The model's performance is greatly impacted by the optimization process. Our study uses the Adam and RMSprop optimization technique to adjust hyper-parameters while changing the epoch and dropout settings. The results showed that, out of all the models, the RMSprop algorithm model with a dropout of 0.5 had the highest accuracy. The Adam algorithm worked better in terms of convergence with a dropout of 0.2. Results showed that the RMSprop's testing phase accuracy was 88.51% based on 150 epochs and a 0.5 dropout. Adam's accuracy was 79.04% with a dropout of 0.2 under the same conditions. Using the RMSprop optimization strategy yields the best training effect for the ResNet-18 pre-training model with and without SE.

#### 3.2.4 Adding more SE blocks to existing model 1

**Figure 17:**
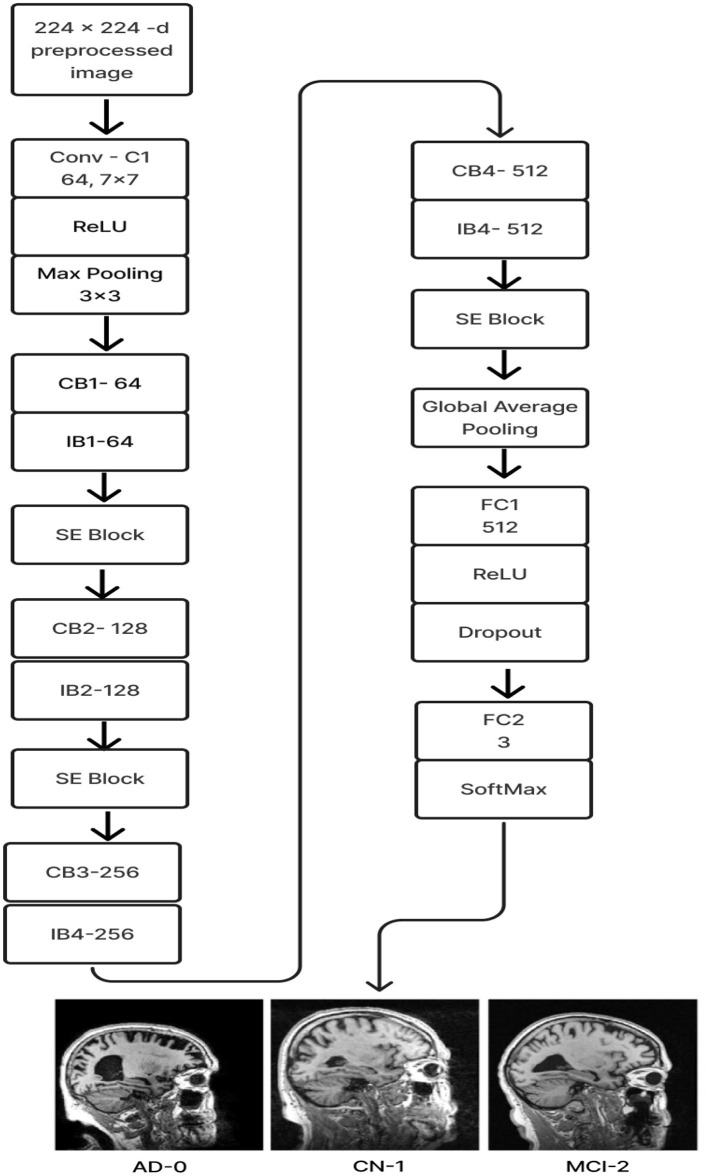
Improvised Model 1 architecture.

**Figure 18:**
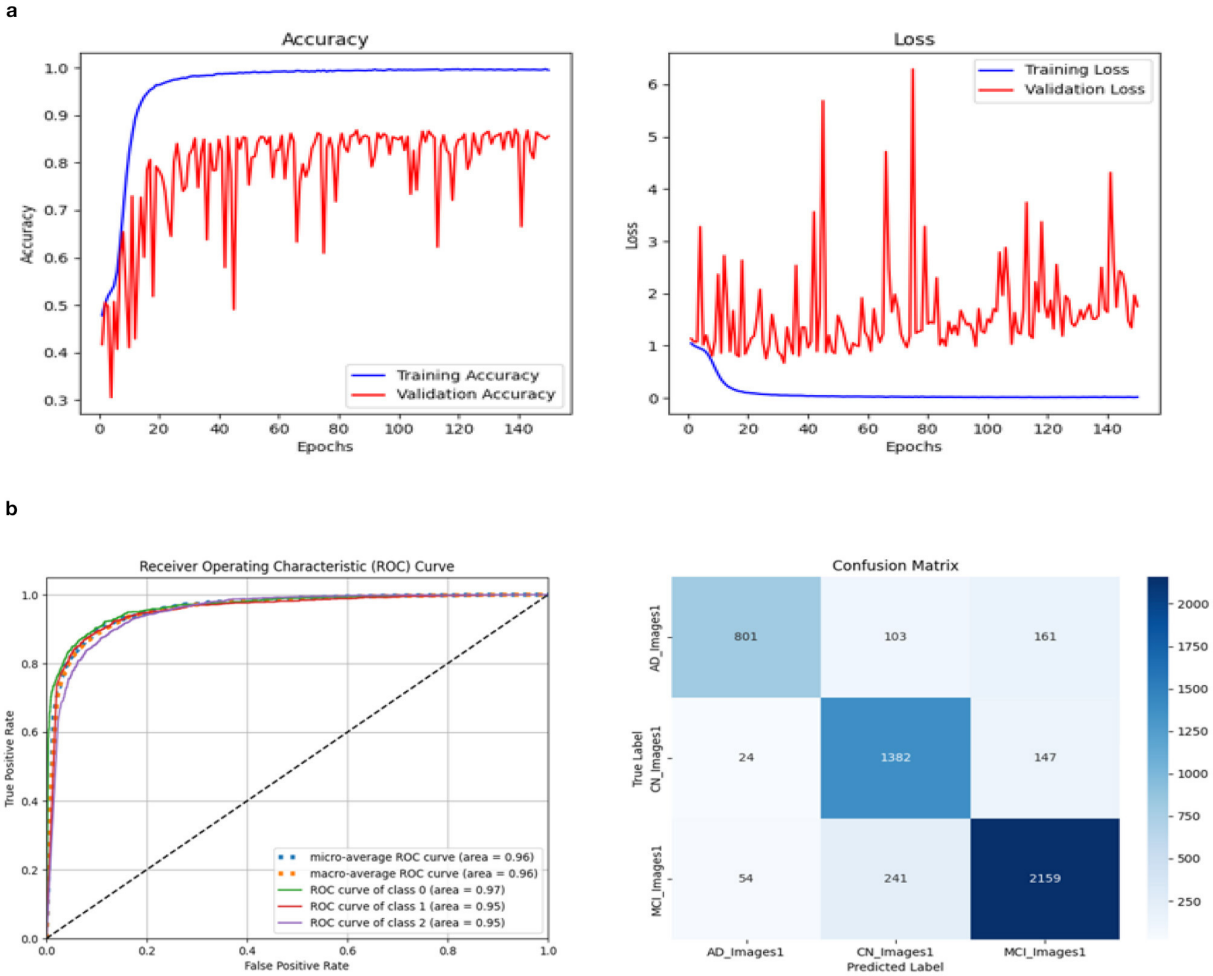
Accuracy and Loss curve of revised Model 1. **(A)** Accuracy and loss. **(B)** ROC curve and Confusion matrix.

Moreover, the model was trained with two more additional SE blocks using the same hyperparameter configurations, resulting in a decrease in training parameters to 6.76 million. The relevant architecture is displayed in Figure 17. The accuracy and loss curve, ROC curve, and Confusion matrix are shown in Figure 18. The Table 7 displays all attained evaluation metric values. Our best test accuracy was 87.05% (139*th* epoch), and our best training accuracy was 99.71% (120*th* epoch). The ROC values for AD, CN, and MCI were 97%, 95%, and 95%, respectively. Compared to the previous one, the training and test accuracies and recall scores were marginally lower. The results were slightly better than the previous one in terms of precision, specificity, F1-score, and ROC.

**Table 7:** Evaluation metric values of revised Model 1.

**Metrics**	**Model 1**	**Improvised Model 1**
Precision	85.83	85.99
Recall	88.51	87.05
Specificity	82.68	84.06
F-Measure	84.68	85.57
Test accuracy	88.51	87.05
Train accuracy	99.81	99.71
ROC values (AD, CN MCI)	95%, 95%,93%	97%, 95%, 95%
Training parameters	9.125 million	6.76 million

#### 3.2.5 Ablation studies

Squeeze and excitation framework, depth-wise convolution, and global average pooling all help the suggested framework reduce training parameters. To assess the effectiveness of the recommended approach, we compared training accuracy, testing accuracy, and ROC values to different patterns of hyperparameter values, such as data augmentation, optimizer, epochs, and dropout. Three architectures were proposed in this section, and predictions are given: (1) discontinuing or putting into practice the transfer learning approach, (2) abandoning or putting into practice data augmentation techniques, (3) including or excluding the Squeeze and Excitation block. Every value is documented in Tables 1–4, 7 and from Figures 6–18 show the impact of the strategies, as mentioned earlier, on prediction performance. The results of F1 measure, ROC values, Precision, Recall, and Specificity indicate that each effort may have a substantial effect on the prediction performance.

### 3.3 Evaluating the models

This section will demonstrate how well the three models work using real-time data and examine how well the suggested model performs using the new OASIS-1 dataset.

#### 3.3.1 Evaluation with real-time patient data

We collected MRI scans in DICOM format and manually recorded demographic data, including Mini-Mental State Examination (MMSE) and Clinical Dementia Rating (CDR), from eight patients in real-time. Due to their low quality, two patients' MRIs were not compatible with the model. First, we converted the DICOM files to JPG format and randomly selected six to nine slices. We followed the entire preprocessing procedure before forecasting each patient's disease using the model and calculated the recall for each model. The suggested Model 1 outperformed the other two models with a score of 100%. Model 2 gave two patients 83.33% and 85.71%. Model 3 yielded a result of 62.5% for one patient. The biomarker threshold values and a thorough examination of real-time data using all three models are displayed in Tables 8, 9, respectively.

**Table 8:** Criteria of bio-markers.

**Bio markers range**					
**MMSE Score**	**CN: 24–30**	**MCI: 18–23**	**AD: 0–17**		
CDR	0: None	0.5: Questionable	1: Mild	2: Moderate	3: Severe

**Table 9:** Evaluation: real-time patient data analysis.

	**Actual**			**Predicted value**			**Recall (%)**		
**Patients**	**MMSE**	**CDR**	**Actual**	**M1**	**M2**	**M3**	**M1**	**M2**	**M3**
Roselin	21	0.5 to 1	MCI	CN:0,MCI: 6,AD:0	CN:1,MCI: 5,AD:0	CN:0,MCI: 6,AD:0	100	83.33	100
Chacko	24	0.5 to 1	MCI	CN:0,MCI: 9,AD:0	CN:0,MCI: 9,AD:0	CN:0,MCI: 9,AD:0	100	100	100
Joseph	26	0.5 to 1	CN/MCI	CN:0,MCI: 7,AD:0	CN:0,MCI: 6,AD:1	CN:0,MCI: 5,AD:2	100	85.71	62.5
Justin	12	1 to 2	AD/MCI	CN:0,MCI: 8,AD:0	CN:0,MCI: 7,AD:1	CN:0,MCI: 7,AD:1	100	100	100
Laila	8	2 to 3	AD/MCI	CN:0,MCI: 7,AD:2	CN:0,MCI: 9,AD:0	CN:0,MCI: 8,AD:1	100	100	100
Narayan	10	2 to 3	AD/MCI	CN:0,MCI: 7,AD:0	CN:0,MCI: 6,AD:1	CN:0,MCI: 7,AD:0	100	100	100
Vasan	22	0.5 to 1	MCI	Unfit	Unfit	Unfit	NA	NA	NA
Dhaya	6	3	AD	Unfit	Unfit	Unfit	NA	NA	NA

#### 3.3.2 Evaluation with OASIS-1 dataset

We randomly selected data from ten patients using the OASIS-1 dataset, including MRI sagittal GIF images and demographic information from CSV files. The GIF images were converted to JPG format and pre-processed. Our model achieved a recall of 90%, with results displayed in Table 10.

**Table 10:** Evaluate the model with OASIS dataset.

**Patients**	**Actual**			**Predicted value by Model 1**
**Subject-ID**	**MMSE**	**CDR**	**Actual**	**Proposed model**
OAS1_0018_MR1	28	0	CN	MCI
OAS1_0056_MR1	15	1	MCI/AD	MCI
OAS1_0098_MR1	18	0.5	AD	AD
OAS1_0179_MR1	21	0.5	MCI	MCI
OAS1_0184_MR1	16	1	AD	AD
OAS1_0185_MR1	17	1	MCI/AD	MCI
OAS1_0308_MR1	15	2	AD	AD
OAS1_0351_MR1	15	2	AD	AD
OAS1_0382_MR1	15	1	AD	AD
OAS1_0430_MR1	17	1	MCI/AD	MCI

## 4 Conclusion

To sum up, we combined the SE block and reduced parameters using Depth-wise convolution to build a Deep CNN_ResNet-18-based model from the ground up. The network's feature extraction and classification sections are integrated after the SE module. The global average pool is selected by the model in order to reduce the overall number of model parameters and speed up convergence. A total of 25,357 MRI brain scans were used in 150 training epochs after data augmentation. The scratch model's accuracy during testing, employing depth-wise convolution and SE, reached 88.51%, with ROC values of AD:95, CN:95, and MCI:93, according to experimental results.

The scratch model with SE has shown the best testing accuracy with RMSprop as an optimizer and a dropout of 0.5. Using the transfer learning approach, one can get faster convergence, higher accuracy, more efficient discrimination across the dataset's numerous classes, and reduced training time compared to designing and training the model from scratch. Among all three models, the pre-trained model ResNet-18 with SE's accuracy is 4.75% higher than the scratch model on the test dataset. In contrast, the other pre-trained model, ResNet-18-without SE's accuracy, is 3.9% higher than the scratch model with SE and depth convolution. Regarding ROC values, the pre-training model with SE shows 3 to 4 points more than the scratch model and 4 to 2 points more than the pre-training model without SE. The suggested approach performed better than the others, as shown by the overall comparison with the state-of-the-art techniques. The results of this study show that methodologies based on deep learning can greatly aid in the identification of neurodegenerative diseases. Using real-time patient data to evaluate all the models suggests that the proposed one could aid in differentiating between the various stages of AD diagnosis. The suggested model fared better when tested using the OASIS dataset as well.

## 5 Limitation and future scope

Inequality of class was one of our main issues. Each of the three classes was in the 1:1.47:2.33 ratio. Additionally, we had problems with the regularity, shape, and intensity variation of a significant number of the MRI images from the ADNI dataset. The center slices were the only ones deemed relevant. Real-time data collection was highly challenging to ethical reasons. Doctors' assistance is still needed to confirm the forecast. Using a series of psychological and mental tests and activities, clinicians manually determine the patients' MMSE and CDR ratings. Each clinician has a different set of results. Therefore, it was necessary to establish certain assumptions in order to test our model against these results. When more SE blocks were added in the hopes of improving precision, the opposite occurred. The performance was deteriorating as increasing the epochs beyond 150. There were more variance in prediction performance and metric values among different optimizers for a particular combination of dropout and epochs. Owing to the intricacy of medical data, the entire training process required a significant amount of processing power to produce consistent results.

Currently, the suggested scratch model solely employs MRI. In the future, additional imaging modalities, such as sMRI, fMRI, rs-fMRI images, PET, etc., can be acquired and tested in the model. We can balance the dataset using a few sophisticated strategies to increase accuracy. We can offer various training and testing options, including using the ADNI for training and additional domain datasets, such as AIBL, for testing. Other advanced image processing techniques can be used to enhance accuracy, and optimized strategies for the pertinent feature selection can help improve the efficiency of feature extraction and raise test accuracy overall.

A few researchers have developed a graph-based network and added hypergraphs to the generative model to fuse multimodal data (rs-fMRI and DTI) to more accurately and robustly predict brain connection abnormalities at different stages of AD. In the future, we might concentrate on a method that uses graphs to solve the same problem. Additionally, we may think about applying eXplainable Artificial Intelligence (XAI) techniques to make sense of the interpretation of the local and global properties that our model's SE block learns. This would provide valuable information on how the model influences AD classification and advance our understanding of its decision-making process (Hazarika et al., 2022a).

## Data Availability

The original contributions presented in the study are included in the article/supplementary material, further inquiries can be directed to the corresponding authors.
